# Molecular determinants of αVβ5 localization in flat clathrin lattices – role of αVβ5 in cell adhesion and proliferation

**DOI:** 10.1242/jcs.259465

**Published:** 2022-06-06

**Authors:** Alba Zuidema, Wei Wang, Maaike Kreft, Onno B. Bleijerveld, Liesbeth Hoekman, Jonas Aretz, Ralph T. Böttcher, Reinhard Fässler, Arnoud Sonnenberg

**Affiliations:** 1 Division of Cell Biology I, The Netherlands Cancer Institute, Plesmanlaan 121, Amsterdam 1066 CX, The Netherlands; 2 Proteomics Facility, The Netherlands Cancer Institute, Amsterdam 1066 CX, The Netherlands; 3 Department of Molecular Medicine, Max Planck Institute of Biochemistry, Am Klopfespitz 18, 82152 Martinsried, Germany

**Keywords:** Flat clathrin lattice, Focal adhesion, Integrin, Vitronectin, Proliferation

## Abstract

The vitronectin receptor integrin αVβ5 can reside in two distinct adhesion structures – focal adhesions (FAs) and flat clathrin lattices (FCLs). Here, we investigate the mechanism that regulates the subcellular distribution of β5 in keratinocytes and show that β5 has approximately 7- and 5-fold higher affinity for the clathrin adaptors ARH (also known as LDLRAP1) and Numb, respectively, than for the talin 1 (TLN1); all proteins that bind to the membrane-proximal NPxY motif of the β5 cytoplasmic domain. Using mass spectrometry, we identified β5 interactors, including the Rho GEFs p115Rho-GEF and GEF-H1 (also known as ARHGEF1 and ARHGEF2, respectively), and the serine protein kinase MARK2, depletion of which diminishes the clustering of β5 in FCLs. Replacement of two serine residues (S759 and S762) in the β5 cytoplasmic domain with phospho-mimetic glutamate residues causes a shift in the localization of β5 from FAs into FCLs without affecting the interactions with MARK2, p115Rho-GEF or GEF-H1. Instead, we demonstrate that changes in the actomyosin-based cellular contractility by ectopic expression of activated Rho or disruption of microtubules regulates β5 localization. Finally, we present evidence that β5 in either FAs or FCLs functions to promote adhesion to vitronectin, cell spreading, and proliferation.

## Abstract (precis)

**Summary:** The mechanism that regulates the subcellular distribution (focal adhesions versus flat clathrin lattices) of integrin β5 in keratinocytes is dependent on the level of tension, MARK2 and its preferred binding to the clathrin adaptors ARH and NUMB over talin.

## INTRODUCTION

Cell adhesion to neighboring cells and/or the surrounding extracellular matrix (ECM) is essential in multicellular organisms for tissue development and homeostasis. Integrins are a major family of transmembrane cell–ECM adhesion receptors that are formed through heterodimerization of an α and a β subunit, and link the intracellular cytoskeleton to ECM components, such as collagens, laminins, fibronectin and vitronectin (Hynes, 2002). The integrin αVβ5 binds to the arginine (R), glycine (G), aspartic acid (D) tri-peptide in vitronectin. This integrin is dispensable for normal mouse development (Huang et al., 2000) but is required for photoreceptor function and viability by regulating retinal adhesion (Nandrot et al., 2006). Additionally, increased vitronectin synthesis and elevated levels of integrin β5 correlate with disease progression of different types of cancer, including colorectal, brain, breast and non-small cell lung cancer (Gladson et al., 1997; Uhm et al., 1999; Bianchi-Smiraglia et al., 2013; Bai et al., 2015; Vogetseder et al., 2013; Schittenhelm et al., 2013; Tomasini-Johansson et al., 1994). It has been shown that integrin β5 plays a role *in vivo* in adhesion and early invasion of metastatic colon carcinoma cells into the liver (Enns et al., 2005) and in tumor growth of breast carcinoma cells (Bianchi-Smiraglia et al., 2013).

Intriguingly, integrin β5 can be found in two distinct adhesion complexes – focal adhesions (FAs) and flat clathrin lattices (FCLs) (Lock et al., 2019; Zuidema et al., 2020; Baschieri et al., 2020). FAs serve as anchorage sites between the ECM and the intracellular actin cytoskeleton and regulate multiple and wide-ranging cellular processes by functioning as bidirectional signaling units (Burridge, 2017). In contrast, the assembly of adhesion complexes containing integrin β5 outside of FAs (Wayner et al., 1991) has recently received more attention, as multiple studies have reported on β5-containing FCLs, clathrin plaques or reticular adhesions (Zuidema et al., 2018; Leyton-Puig et al., 2017; Lock et al., 2018; Baschieri et al., 2018), which all refer to the same type of structure (Lock et al., 2019). Integrin β5-containing FCLs are structurally and dynamically different from the canonical clathrin pits (Baschieri et al., 2020; Grove et al., 2014; Lampe et al., 2016) and FAs (Lock et al., 2019; Zuidema et al., 2020). They have been proposed to form as a consequence of frustrated clathrin-mediated endocytosis and might mediate cell–ECM adhesion during mitosis (Lock et al., 2018), serve as platforms for cytoskeletal anchorage in skeletal muscle (Franck et al., 2019), and promote growth factor receptor signaling (Leyton-Puig et al., 2017; Baschieri et al., 2018). The assembly of integrin β5 in FCLs is characteristic for a wide range of cell types and a single cell can contain both β5-containing FCLs and FAs (Lock et al., 2018).

Previously, our laboratory investigated the mechanisms controlling the assembly of β5-containing FCLs in keratinocytes and demonstrated that binding of the cytoplasmic domain of β5 by the clathrin adaptor proteins ARH (also known as LDLRAP1), Numb, EPS15 and EPS15L1 is required for FCL formation (Zuidema et al., 2018). However, how the subcellular localization of integrin β5 in FAs versus FCLs contributes towards its function is still unclear. In this study, we further analyzed the molecular mechanisms that control the assembly of β5-containing adhesion complexes in more detail and demonstrate that the integrin β5 cytoplasmic domain binds more strongly to ARH and Numb than to talin 1 (hereafter talin), and displays only very low binding to kindlin-1 or -2 (kindlin-1/2; also known as FERMT1 and FERMT2). Furthermore, phospho-mimetic mutation of two serine residues (S759/762) in a β5-SERS motif, which has been implicated in controlling cell attachment and migration (Li et al., 2010), promotes localization in FCLs. We also report that destabilization of microtubules promotes relocalization of integrin β5 from FCLs into FAs through modulation of tension. Finally, we present evidence that β5 mediates adhesion and promotes cell proliferation regardless of its localization in FAs or FCLs.

## RESULTS

### Localization of integrin β5 in human cancer cells

**Fig. 1.**
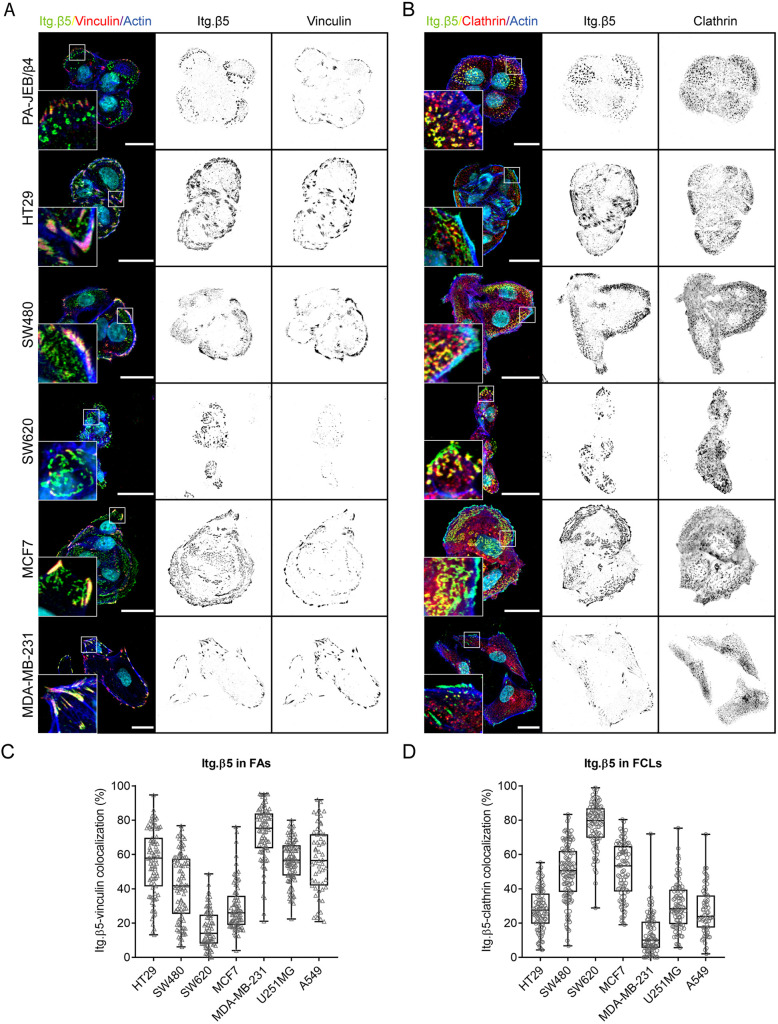
**Subcellular distribution of integrin β5 in keratinocytes and multiple cancer cells.** (A,B) Immunofluorescence images showing integrin β5 (Itg. β5; green in merge), vinculin (A) or clathrin (B) (red in merge), actin (blue), DAPI (cyan). Scale bars: 20 μm. (C,D) Quantifications of integrin β5 colocalization with vinculin in FAs (C) or with clathrin in FCLs (D). Data were obtained from three independent experiments. Total cells analyzed per condition: HT29=86 (C) and 94 (D), SW480=89 (C) and 115 (D), SW620=70 (C) and 90 (D), MCF7=82 (C) and 88 (D), MDA-MB-231=79 (C) and 89 (D), U251MG=97 (C) and 88 (D), A549=55 (C) and 63 (D). Representative images of U251MG and A549 cells are shown in Fig. S1. Box plots range from the 25th to 75th percentile; central line indicates the median; whiskers show smallest to largest value.

The level of integrin β5, which can reside in two distinct adhesions in a variety of cell lines (Table S1), has been correlated with disease progression of colorectal, brain, breast and non-small cell lung cancer (Gladson et al., 1997; Uhm et al., 1999; Bianchi-Smiraglia et al., 2013; Bai et al., 2015; Vogetseder et al., 2013; Schittenhelm et al., 2013; Tomasini-Johansson et al., 1994). Because the subcellular localization of proteins plays an important role in the regulation of cell function, we investigated the localization of β5 in a panel of human cancer cell lines derived from colorectal (HT29, SW480, and SW620), brain (U251MG), breast (MCF7 and MDA-MB-231) and lung (A549) cancers (Fig. 1; Fig. S1). Similar to PA-JEB/β4 and HaCaT keratinocytes (Zuidema et al., 2018), β5 localizes almost exclusively in FCLs in SW620 cells. In MCF7 cells, β5 can be found in both FAs and FCLs, but localization in FCLs is favored. MDA-MB-231 cells hardly form β5-containing FCLs. Integrin β5 can be found predominantly in FAs in HT29, U251MG and A549 cells. In SW480 cells, β5 is distributed roughly equally between FAs and FCLs (Fig. 1; Fig. S1). Based on these observations, we conclude that the localization of β5 is independent of tissue origin.

### Selective binding of ARH by integrin β5

**Fig. 2.**
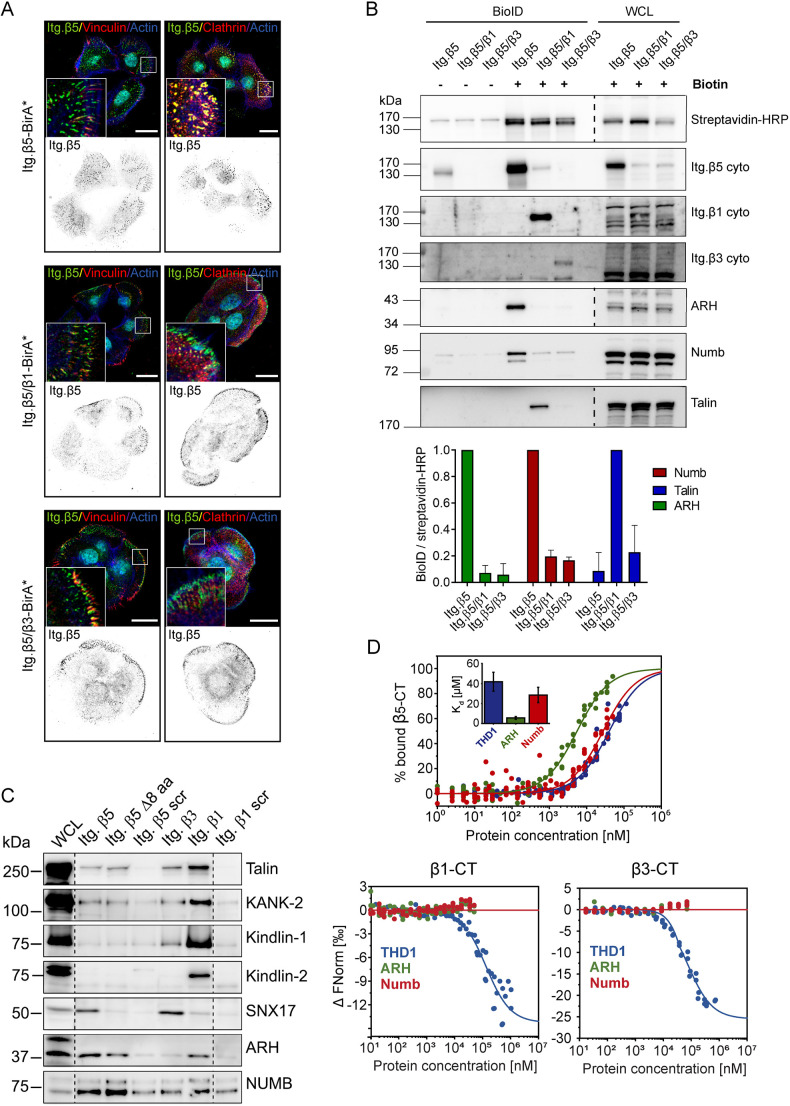
**Adaptor protein binding to integrin β subunits.** (A) Colocalization of integrin chimeras fused to the promiscuous biotin ligase BirA* (green in merge) with vinculin (red; left panels) or clathrin (red; right panels) in PA-JEB/β4 keratinocytes. Actin is shown in blue. Nuclei are stained with DAPI (cyan). Scale bars: 20 μm. (B) Representative western blots of BioID assays performed using the integrin chimeras shown in A. Quantifications of ARH, Numb and talin signal intensities normalized to streptavidin-HRP levels are shown (*n*=3; bars show mean±s.d.). (C) Representative western blots of pulldown assays (from two repeats) using synthetic integrin β cytoplasmic domains in RAC-11P cell lysates. (D) MST assay demonstrating binding of ARH, Numb, and talin-1 head domain (THD1) peptides to the β5 cytoplasmic domain (*n*≥5). Inset in top panel shows calculated *K*_d_ (mean±s.d.). β5-CT, β5 cytoplasmic tail; β5 Δ8 aa, β5 mutant carrying a deletion of a stretch of 8 amino acids (Val^783^–Phe^790^) located between the NPxY and NxxY motifs in the cytoplasmic domain of β5; Itg. β5/β1, chimeric receptor containing the extracellular and transmembrane domain of β5 and the cytoplasmic domain of β1; Itg. β5/β3, chimeric receptor containing the extracellular and transmembrane domain of β5 and the cytoplasmic domain of β3; scr, scrambled; WCL, whole-cell lysates.

To understand the mechanism by which integrin β5 uniquely localizes in two types of adhesion complexes, we compared the sequence of and adaptor proteins associated with integrin β5 versus β1 and β3. All three integrin subunits contain a highly conserved membrane proximal (MP) NPxY motif that serves as a canonical binding site for the talin phospho-tyrosine-binding (PTB)/4.1 protein, ezrin, radixin and moesin (FERM) domain (from now on referred to as the talin head domain; THD) and the PTB domain-containing clathrin adaptor proteins ARH, Numb and Dab2 (Calderwood et al., 2003; Zuidema et al., 2018). We hypothesized that differential binding between the integrin β1, β3 and β5 cytoplasmic domains and the PTB-containing adaptor proteins could contribute to the distinct subcellular distribution pattern of these integrins. To test this, we generated integrin chimeras fused to a promiscuous biotin ligase (BirA*) to perform proximity biotinylation (BioID) experiments with PA-JEB/β4 keratinocytes grown for 1 day in DMEM with fetal calf serum (FCS) (Roux et al., 2012). Similar to the integrin β5^ex^/β1^in^ and β5^ex^/β3^in^ (ex, extracellular; in, intracellular) chimeras previously described (Zuidema et al., 2018), the integrin β5^ex^/β1^in^- and β5^ex^/β3^in^–BirA* fusion proteins preferentially localize in FAs (Fig. 2A). BioID experiments with subsequent western blot analysis showed strong biotinylation of the clathrin adaptor proteins ARH and Numb by β5–BirA*, but not by the β5^ex^/β1^in^– and β5^ex^/β3^in^–BirA* fusion proteins. Furthermore, the β5–BirA* construct was unable to biotinylate talin, in stark contrast to the β5^ex^/β1^in^–BirA* chimera (Fig. 2B). We further validated these findings by performing pulldown experiments with integrin β1, β3 and β5 cytoplasmic domain peptides. Again, we detected an interaction between β5 and ARH and Numb and between β1 and the FA proteins talin, KANK-2 and kindlin-1/2 (Fig. 2C). Microscale thermophoresis (MST) measurements confirmed the binding of β5 to ARH and showed that the affinity of the cytoplasmic tail peptide of β5 for ARH (*K*_d_=5.6±1.4 µM; mean±s.d.) is higher than that for Numb (*K*_d_=28.5±7.7 µM) and the talin-1 head domain (THD1) (*K*_d_=41.7±9.5 µM), whereas no binding of β1 and β3 to ARH and Numb was detected in the concentration range tested (up to 50 µM for β1 and 75 µM for β3; Fig. 2D). Together, these experiments demonstrate that the cytoplasmic domain of integrin β5 confers a unique ability to bind to the clathrin adaptor proteins ARH and Numb, despite the presence of the highly conserved MP-NPxY and MD-NxxY motifs present in the cytoplasmic domains of both integrin β1 and β3.

Integrin β5 differs from β1 and β3 by the presence of an 8-amino-acid insertion between the MP-NPxY and MD-NxxY motifs, which might be responsible for the differential binding between the integrin β1, β3 and β5 cytoplasmic tails with the clathrin adaptors. However, deletion of the β5 8-amino-acid sequence prevented neither the clustering of β5 in FCLs (Zuidema et al., 2018) nor the binding of cytoskeletal or clathrin adaptor proteins to the β5 tail (Fig. 2C). On the other hand, the binding of β5 to SNX17 was abrogated by the deletion (Fig. 2C).

Interestingly, in contrast to integrin β1 and β3, we detected no interaction between β5 and kindlin-1/2 in our peptide pulldown experiments (Fig. 2C) and only a low affinity interaction by MST (Fig. S2A). THD1 bound with a slightly higher affinity to the integrin β5 subunit compared to β1 and β3 *in vitro* (Fig. S2A), which is in agreement with reported affinities of talin for β1 and β3 (Anthis et al., 2010). Similar to talin, kindlin-1/2 can also connect integrins to the actin cytoskeleton. We hypothesized that the reduced binding of kindlin-1/2 to β5 as compared to that seen with β1 and β3 could contribute to its localization in FCLs versus FAs. To test this, we introduced a disruptive Y>A mutation in the MD-NxxY motif responsible for kindlin-1/2 binding in the β5^ex^/β3^in^ chimera. This construct (Itg. β5/β3^Y786A^) localized predominantly in FAs, similar to the wild-type β5^ex^/β3^in^ chimera (Fig. S2B), indicating that the absence of kindlin-1/2 binding does not influence integrin β5 localization.

The integrin β1, β3 and β5 subunits also differ in charged residues adjacent to the membrane-proximal NPxY motif. Changing the charged into noncharged residues, and vice versa, at the −5 and +2 position relative to the tyrosine residue of the β3 NPxY motif has been shown to inhibit PTB domain binding (Calderwood et al., 2003). Integrin β5 contains a positively charged residue at position +2 relative to the tyrosine of its NPxY motif, in contrast to an uncharged or a negatively charged residue for β1 and β3, respectively (Fig. S3A). Another striking difference between the amino acid sequences of the integrin tails is the tyrosine at position −8 in β5 versus the tryptophan present at the corresponding position in β1 and β3 (Fig. S3A). We wondered whether these residues could play a role in determining the subcellular distribution of β5, potentially through regulating the binding affinity of ARH. To this end, we introduced β5 mutants containing Y766W, both K776E and P777A, or a 12-amino-acid substitution of residues 766–777 to mimic the sequence of integrin β3 in β5-deficient keratinocytes. However, none of these β5 mutants displayed an altered localization compared to the wild-type integrin (Fig. S3A,B).

### Serine residues 759 and 762 regulate integrin β5 localization

**Fig. 3.**
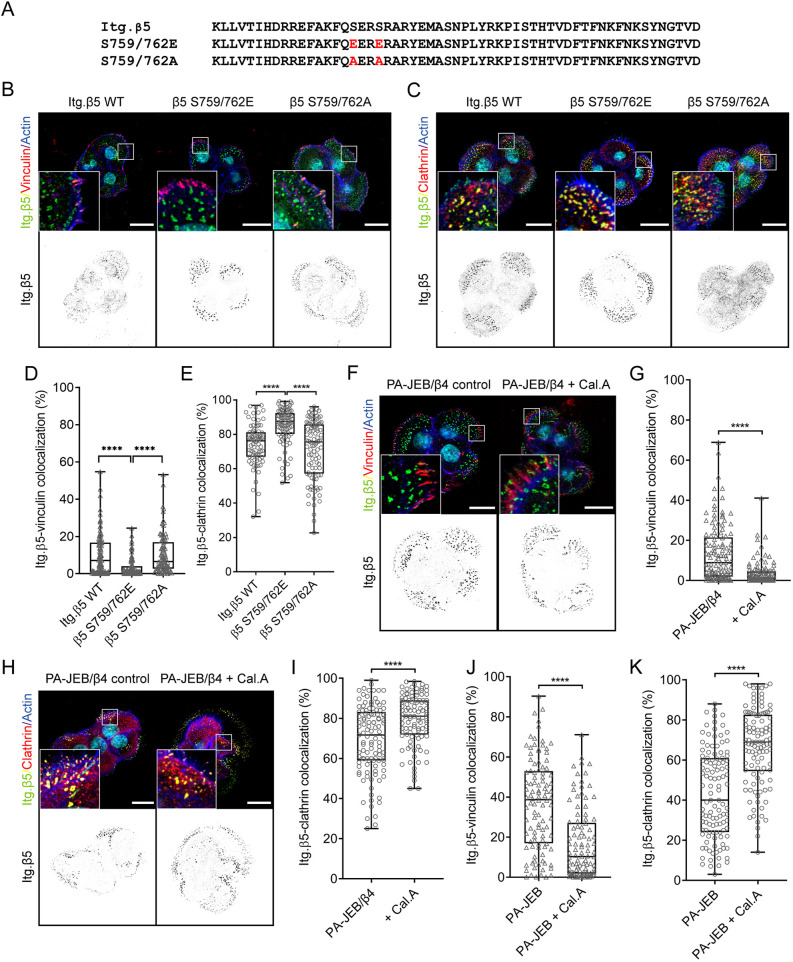
**Serine 759 and 762 are involved in regulating the localization of integrin β5.** (A–C) Integrin β5 containing S759/762E or S759/762A mutations (A) were expressed in β5-deficient PA-JEB/β4 keratinocytes and the subcellular distribution of β5 was compared to β5-deficient keratinocytes expressing wild-type (WT) β5. Merged images show integrin β5 (green), vinculin (B) or clathrin (C) (red), actin (blue) and the cell nuclei (cyan). (D,E) Analysis of wild-type versus mutant integrin β5 clustering in FAs or FCLs. (F–I) PA-JEB/β4 keratinocytes were grown in 10% FCS-supplemented DMEM culture medium overnight and then treated with 5 nM calyculin A (Cal.A) or DMSO (vehicle control) for 30 min prior to fixation. Merged images show integrin β5 (green), vinculin (F) or clathrin (H) (red), actin (blue) and the cell nuclei (cyan), quantifications of β5 clustering in FAs or FCLs are shown in G,I. (J,K) Analysis of integrin β5 clustering in FAs or FCLs in PA-JEB keratinocytes after treatment with Cal.A. Representative confocal microscopy images are shown in Fig. S3. Scale bars: 20 μm. Data were obtained from three independent experiments. Total cells analyzed per condition: 109 (WT), 116 (S>E), 103 (S>A) (D), 81 (WT), 77 (S>E), 101 (S>A) (E), 126 and 123 (G), 111 and 101 (I), 106 and 102 (J), 115 and 109 (K). ****P<0.0001 (Mann–Whitney *U*-test). Box plots range from the 25th to 75th percentile; central line indicates the median; whiskers show smallest to largest value.

The amino acid sequence Ser-Glu-Arg-Ser (SERS) is another region of the β5 cytoplasmic domain that we hypothesized could influence its localization. The serine residues in this stretch of amino acids are phosphorylated by p21-activated kinase 4 (PAK4) (Li et al., 2010; Zhang et al., 2002) and PAK4 binding to the phosphorylated residues accelerated integrin β5 turnover within lamellipodial structures (Li et al., 2010; Zhang et al., 2002). To investigate whether the β5-SERS could play a role in regulating the localization of β5, we generated β5-deficient keratinocytes expressing a β5-SERS phospho-mimetic (S759E/S762E; hereafter S759/762E) or phospho-inhibitory (S759A/S762A; hereafter S759/762A) mutant (Fig. 3A). We observed reduced localization of the phospho-mimetic integrin β5-S759/762E mutant in FAs and increased localization in FCLs, as indicated by reduced colocalization with vinculin and increased colocalization with clathrin. No significant difference in localization to either FAs or FCLs was observed with the β5-S759/762A mutants (Fig. 3B–E). To further validate whether phosphorylation of the SERS region regulates β5 localization, we treated PA-JEB/β4 keratinocytes with calyculin A, a potent inhibitor of phosphatase-1 and -2A. Similar to the β5-S759/762E-expressing cells, treatment with calyculin A decreased the localization of integrin β5 in FAs of PA-JEB/β4 keratinocytes (Fig. 3F–I). We repeated this experiment with PA-JEB keratinocytes, which lack β4 and in which β5 is found at higher abundance in FAs than in PA-JEB/β4 cells (Wang et al., 2020). In line with our previous findings, calyculin A treatment reduced the localization of integrin β5 in FAs and increased the localization in FCLs (Fig. 3J,K; Fig. S3C,D).

**Fig. 4.**
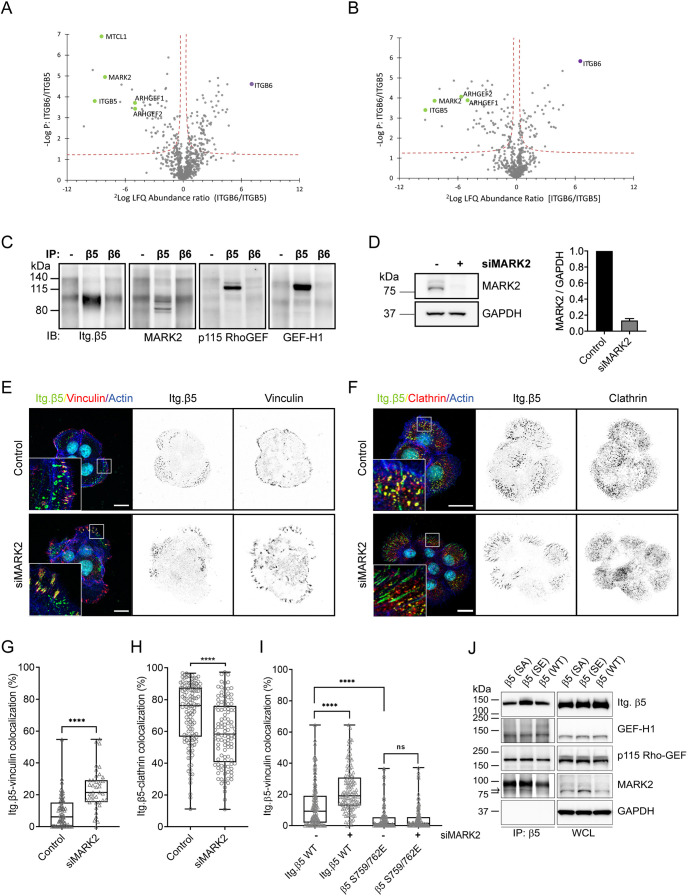
**MARK2 regulates the localization of integrin β5.** (A,B) Immunoprecipitations of integrin β5 and β6 were performed using 5HK2 and 5HK1 antibodies, respectively, in PA-JEB/β4 (A) or HaCaT (B) keratinocytes. Volcano plots show proteins enriched in integrin β5 versus β6 samples. The logarithmic ratio of protein LFQs were plotted against negative logarithmic *P*-values of a two-sided two samples *t*-test. The hyperbolic curve separates significantly enriched proteins from common binders (FDR, 0.05; *n*=3). Proteins discussed here are highlighted in green. (C) Western blots (IB) of normal rabbit serum (−) and integrin β5 and β6 immunoprecipitations (IP) to validate some of the β5 interactors identified by mass spectrometry. MARK2 isoforms have a molecular mass of 77–88 kDa. Representative of two repeats. (D) Representative western blot showing siRNA-mediated knockdown of MARK2 (siMARK2) in PA-JEB/β4 keratinocytes. Quantifications of signal intensities normalized to GAPDH levels are shown (*n*=3; bars show mean±s.d.). Western blots of MARK2 expression were performed in parallel to the immunofluorescence analysis shown in E–H. (E–H) Analysis of integrin β5 clustering in FAs (E,G) or FCLs (F,H) in control versus MARK2-depleted PA-JEB/β4 keratinocytes. Merged images show integrin β5 (green), vinculin (E) or clathrin (F) (red), actin (blue) and the cell nuclei (cyan). Scale bars: 20 μm. Quantifications of β5 clustering in FAs or FCLs are shown in G,H. Data were obtained from three independent experiments. Total cells analyzed per condition: 60 (control), 40 (siMARK2) (G), 122 (control), 105 (siMARK2) (H). (I) Quantifications of integrin β5 wild-type versus β5-S759/762E clustering in FAs. Data were obtained from three independent experiments. Total cells analyzed per condition: 136 [wild type (WT), control], 125 (WT, siMARK2), 118 (S759/762E, control), 140 (S759/762E, siMARK2). ****P<0.0001; ns, not significant (Mann–Whitney *U* test). Box plots range from the 25th to 75th percentile; central line indicates the median; whiskers show smallest to largest value. (J) Western blots of integrin β5 immunoprecipitations to validate if previously defined β5 interactors, MARK2, GEF-H1 and p115-Rho-GEF still associate with wild-type and integrin β5 mutants. The black arrow indicates the position of immunoprecipitated MARK2. Representative of three repeats. SE, S759/762E; SA, S759/762A; WCL, whole-cell lysate.

To identify a kinase that perhaps could be responsible for the β5-S759/S762 phosphorylation, we used a matched set of rabbit serum containing antibodies against β5 (5HK2) or β6 (5HK1; negative control) to immunoprecipitate these integrin subunits and analyzed the interacting proteins by mass spectrometry (Fig. 4A,B; Tables S2 and S3). In contrast to previous studies (Li et al., 2010; Zhang et al., 2002), PAK4 was not found as a significant interactor of integrin β5. The microtubule affinity-regulating kinase 2 (MARK2; also known as Par1b) was the only serine/threonine-protein kinase identified as a significant interactor of β5 in both PA-JEB/β4 and HaCaT keratinocytes (Fig. 4A,B). In addition, p115-RhoGEF (also known as ARHGEF1) and GEF-H1 (also known as ARHGEF2) and multiple proteins that associate with the microtubule network were identified as β5 interactors, including kinesin-1 heavy (KIF5B) and light chain (KLC1) (Kaneko et al., 2020; Jiang et al., 2019), EB1 (also known as MAPRE1) (Askham et al., 2002), HOOK2 (Walenta et al., 2001; Krämer and Phistry, 1996) and NUDC (Aumais et al., 2003, 2001) (Fig. 4A,B; Tables S2 and S3). The MARK2-interacting protein microtubule crosslinking factor 1 (MTCL1) (Sato et al., 2013) was found as a significant interactor of β5, but only in PA-JEB/β4 cells (Fig. 4A). The interactions between β5 and MARK2, p115-RhoGEF and GEF-H1 were validated by co-immunoprecipitation and western blot analysis (Fig. 4C). To assess whether MARK2 could play a role in regulating the subcellular distribution of β5, we depleted MARK2 in PA-JEB/β4 cells using siRNAs and determined the localization of β5 in FAs and FCLs (Fig. 4D–H). MARK2 depletion resulted in a significant increase of β5 clustering within FAs with a concomitant decrease within FCLs (Fig. 4D–H). The same effect was also observed in β5-deficient PA-JEB/β4 cells expressing the wild-type β5 subunit but not for the β5-S759/762E mutant (Fig. 4I), suggesting that MARK2 could promote the localization of β5 in FCLs through binding and phosphorylation of S759 and/or S762. However, binding of MARK2 to β5 was not disrupted when the two serine residues were mutated to alanine residues, or to glutamic acid residues to mimic phosphoserines (Fig. 4J). These mutations also had no effect on the binding of β5 to GEF-H1 or p115Rho-GEF.

We conclude that the β5-SERS region plays a role in the subcellular distribution of the integrin, as the phospho-mimetic replacement of residues 759 and 762 with glutamate residues reduces the clustering of β5 in FAs.

### High cellular tension corresponds with β5 location to FAs

**Fig. 5.**
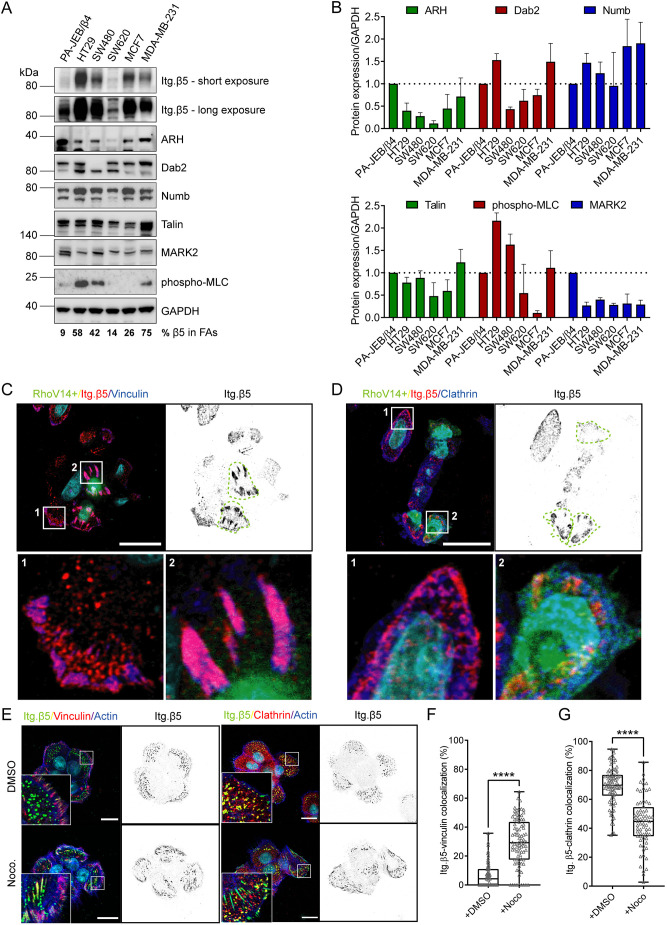
**Actomyosin contractility regulates integrin β5 subcellular distribution.** (A) Representative western blots of integrin adaptor proteins, MARK2, and phosphorylated myosin light chain (MLC) in PA-JEB/β4 keratinocytes, colorectal cancer cells (HT29, SW480, SW620) and breast cancer cells (MCF7, MDA-MB-231). The medians of the percentage values of β5 in FAs (quantified from Figs 1C, 3G) are indicated below. (B) Quantifications of signal intensities normalized to GAPDH levels are shown (*n*=3; bars show mean±s.d.). (C,D) SW480 transfected with RhoV14 (constitutively active) mutant. Merged images show RhoV14 positive cells in green, integrin β5 (red), and vinculin (C) or clathrin (D) (blue), and the cell nuclei (cyan). RhoV14+ cells are indicated with a green dashed line in the integrin β5 channel. Scale bars: 20 μm. (E–G) Analysis of integrin β5 clustering in FAs or FCLs in DMSO (control) versus nocodazole-treated PA-JEB/β4 keratinocytes. (E) Merged images show integrin β5 (green), vinculin or clathrin (red), actin (blue) and the cell nuclei (cyan). Scale bars: 20 μm. (F,G). Quantification of β5 clustering in FAs or FCLs. Data were obtained from three independent experiments. Total cells analyzed per condition: 60 (DMSO) and 75 (Noco) (F), and 92 (DMSO) and 75 (Noco) (G). ****P<0.0001 (Mann–Whitney U test). Box plots range from the 25th to 75th percentile; central line indicates the median; whiskers show smallest to largest values.

So far, we learned that cellular tension (Wang et al., 2020; Zuidema et al., 2018), the serine residues in the β5-SERS region, and the MARK2 and clathrin adaptor proteins, which associate with the β5 cytoplasmic tail, contribute to the assembly of distinct β5-containing adhesion complexes. We wondered whether one of these factors could be the main determinant of β5 clustering in FAs versus FCLs. To this end, we compared the expression levels of talin, MARK2, and clathrin adaptor proteins ARH, Numb and Dab2, and phosphorylation of myosin light chain on serine 19 [phospho-myosin light chain 2 (MCL2)] as an indicator of cellular tension, by western blot in keratinocytes and several colorectal and breast cancer cell lines (Fig. 5A,B). In the colorectal cancer cell lines, the highest level of phospho-MLC was detected in HT29 cells, in which the majority of β5 localizes in FAs, whereas SW620 cells, which mainly form β5-containing FCLs, exhibited the lowest level of phospho-MLC. SW480 cells displayed a roughly equal distribution of β5 in FAs versus FCLs and indeed showed an intermediate level of phospho-MLC. A similar trend was observed for the breast cancer cells MCF7 and MDA-MB-231 (Figs 1 and 5A,B). Thus, a connection between the phospho-MLC levels and the subcellular distribution pattern of integrin β5 could be observed for both colon and breast cancer cell lines. However, when all the colon and breast cancer cell lines were taken together, this relationship was less clear. To confirm that cellular tension also regulates the β5 subcellular distribution in SW480 cells, we transfected these cells with a constitutively active RhoA mutant (RhoV14) and observed that β5 localized almost exclusively in FAs in these cells (Fig. 4C,D), in line with our previous findings in keratinocytes (Wang et al., 2020; Zuidema et al., 2018).

The finding that GEF-H1 is a major integrin β5 interactor, raised the possibility that the microtubule network is involved in the regulation of β5 localization. To test this possibility, we treated PA-JEB/β4 cells with nocodazole and determined the localization of β5 within FAs and FCLs. Disruption of microtubules resulted in a significant shift in the localization of β5 from FCLs to FAs (Fig. 5E–G). Since microtubule disruption has been shown to enhance contractility (Fig. S4A) through activating the RhoA-specific GEF activity of GEF-H1 (Chang et al., 2008), the redistribution of integrin β5 could be a result of GEF-H1 activation following release from destabilized microtubules. We further tested whether the phospho-mimetic or phospho-inhibitory integrin β5 mutant would respond to nocodazole and found neither of their localizations were altered by nocodazole treatment, suggesting these mutations impaired the ability of integrin β5 to sense the change on contractility induced by microtubule interruption.

Because it has been shown that MARK2 can phosphorylate GEF-H1 at S886 and inhibit the RhoA-specific GEF activity (Yamahashi et al., 2011), we depleted MARK2 in PA-JEB and PA-JEB/β4 keratinocytes. Surprisingly, we found that rather than decreasing GEF-H1 phosphorylation, the depletion of MARK2 increased the level of GEF-H1 phosphorylation in both PA-JEB and PA-JEB/β4 keratinocytes (Fig. S4F). Likewise, we observed an increase in the phosphorylation of GEF-H1 after treatment of these cells with calyculin A (Fig. S4F). These data indicate that in PA-JEB and PA-JEB/β4 keratinocytes MARK2 does not regulate the localization of integrin β5 through phosphorylation of GEF-H1 at S886.

In summary, the amount of cellular tension is indicative of the localization of integrin β5, and this could be modulated by microtubule dynamics and GEF-H1.

### Integrin β5 promotes cell proliferation both in FAs and FCLs

**Fig. 6.**
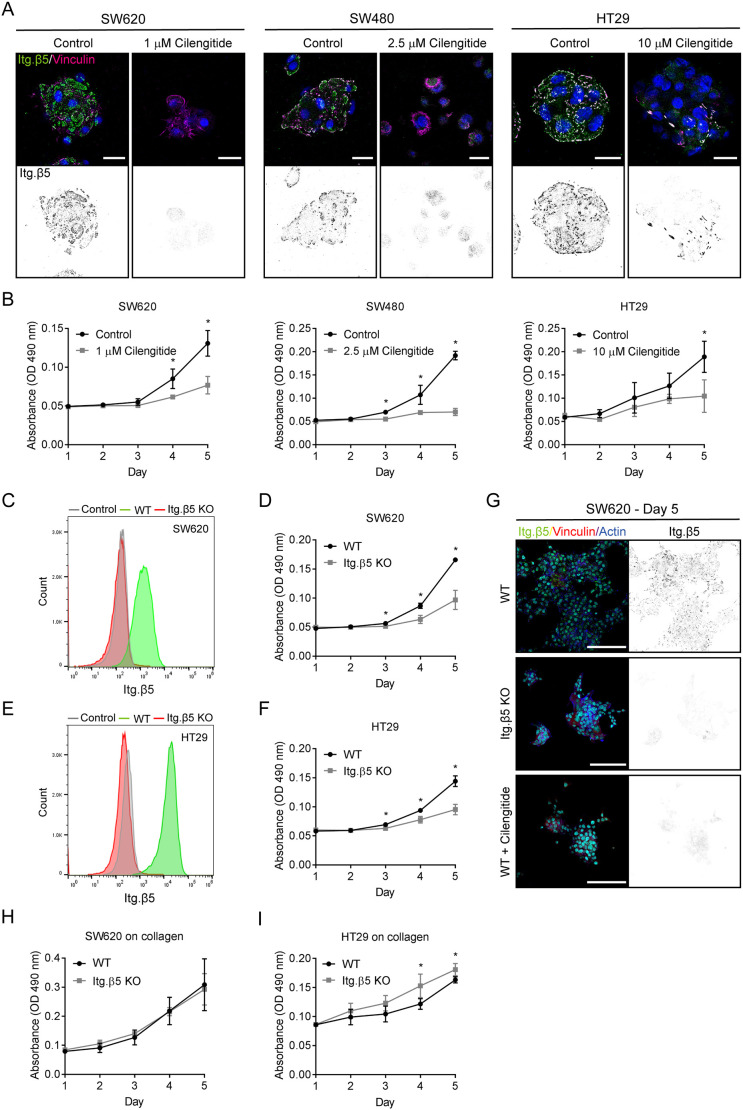
**Integrin β5 promotes colorectal cancer cell proliferation.** (A) Inhibition of β5 clustering by cilengitide treatment in the indicated cell lines. Cells were fixed after 3 days of treatment and β5 (green in merge) and vinculin (magenta) were visualized using confocal microscopy. Cell nuclei are shown in blue. Scale bars: 20 μm. (B) Cells were seeded on day 0 and proliferation was measured from day 1–5 in cells with or without cilengitide (added at day 1). (C,E) FACS plots showing the expression of β5 in SW620 (C) and HT29 (E) wild-type (WT) and β5 knockout cells. Cells stained with a secondary PE-conjugated antibody only were used as negative control (*n*=2). (D,F) Proliferation of SW620 and HT29 wild-type and β5 knockout cells. (G) Representative IF images showing integrin β5 (green in merge), vinculin (red), actin (blue), nuclei (cyan) in SW620 wild-type, β5-deficient, and cilengitide-treated cells that were cultured for 5 days on coverslips prior to fixation. Scale bars: 100 μm. (H,I) Proliferation of SW620 (H) and HT29 (I) wild-type and β5 knockout cells on 3.2 μg ml^−1^ collagen. Results shown represent mean±s.d. of three biological replicates, of which each experiment was performed in triplicate. *P<0.05 (two-sided unpaired Student's *t*-test).

The integrin β5 plays a role in breast cancer and osteosarcoma cell proliferation (Bianchi-Smiraglia et al., 2013; Lock et al., 2018). Here, we analyzed the role of integrin β5 in cell proliferation of SW620, HT29 and SW480 colorectal cancer cells, in which β5 localizes predominantly in FCLs, FAs or is distributed equally in both adhesions, respectively. Because none of these cell lines express the integrin β3 subunit (Fig. S5A), we used the integrin αVβ3/αVβ5 antagonist cilengitide as a tool to inhibit αVβ5 function. The optimal concentration for inhibition was determined for each cell line by assessing the ability of cilengitide to inhibit the clustering of β5 (Fig. 6A). Cell proliferation was determined by Crystal Violet staining, and revealed a significant decrease in proliferation of all three cell lines after inhibition of β5 with cilengitide (Fig. 6B). This result was confirmed using integrin β5-knockout SW620 and HT29 cell lines, which also showed a decreased proliferation compared to the wild-type cells (Fig. 6C–F). From these results, we conclude that integrin β5 promotes colorectal cancer cell proliferation regardless of its localization in FCLs or FAs.

Remarkably, inhibition of vitronectin binding by inhibition or deletion of integrin β5 resulted in a drastic change in phenotype, as we observed that β5-deficient and cilengitide-treated SW620 cells grew on top of each other, in contrast to the control cells, which mainly formed a 2D monolayer (Fig. S5B; Fig. 6G). Possibly, the lack of cell adhesion to vitronectin led to the formation of more and/or stronger cell–cell contacts and subsequent growth of cells in three dimensions. Knockout of integrin β5 in HT29 cells also produced a dramatic change in phenotype; after 5 days in culture these cells showed reduced spreading with no actin stress fibers or FAs, and were more scattered compared to the wild-type cells (Fig. S5C,D). These phenotypical changes could be rescued to some extent by re-expression of integrin β5 (Fig. S5D,E). Unfortunately, the integrin β5 rescued cell lines displayed very high intracellular levels of β5 and therefore did not exactly resemble the wild-type cells (Fig. S5F). We wondered whether the changes in cell proliferation and morphology upon inhibition or deletion of integrin β5 could be driven by altered intracellular signaling pathways or by defective adhesion. Comparing the levels of phospho-Akt (antibody recognizes phosphorylated Akt1–Akt3), phospho-MAPK (phospho-ERK1/2; ERK1 and ERK2 are also known as MAPK3 and MAPK1, respectively), and phospho-FAK (FAK is also known as PTK2) in HT29 and SW620 wild-type, β5-deficient and rescue cells (Fig. S5F) revealed no obvious changes in phospho-Akt or phospho-MAPK levels between integrin β5-deficient and -proficient cells. The β5-deficient HT29 cells exhibited lower levels of phospho-FAK than their wild-type form; however, phospho-FAK levels remained low in β5 rescue cells. In addition, we made use of U251MG cells, which have a subcellular β5 distribution comparable to that of HT29 (Fig. S1), and observed reduced phospho-FAK levels in β5-deficient U251MG cells (Fig. S5F). Unfortunately, the U251MG rescued cell line also showed very high intracellular levels of integrin β5, and thus could not be considered as a proper control in this experiment (Fig. S5F). Nevertheless, we can conclude that deletion of β5 hardly affects Akt and MAPK signaling in HT29, SW620 and U251MG cells. Deletion of β5 might result in reduced phosphorylation of FAK, which seems more prominent in cells in which β5 localizes in FAs. Because we did not observe disrupted signaling pathways in both SW620 and HT29 β5-deficient cells, we hypothesized that the impaired proliferation of these cell lines is most likely caused by defective adhesion to and spreading on vitronectin. Indeed, the proliferation rate of integrin β5-deficient SW620 cells was identical to wild-type cells when cells were grown on collagen-coated plates (Fig. 6H). Similar results were obtained for wild-type and integrin β5-deficient HT29 cells grown on collagen, where the β5-deficient cells even grew slightly faster than the wild-type cells (Fig. 6I).

Taken together, integrin β5 promotes colorectal cancer cell proliferation by mediating adhesion and cell spreading on vitronectin-coated surfaces and plays this role regardless of its localization in FAs or FCLs.

### FAs form first during early cell–ECM adhesion

**Fig. 7.**
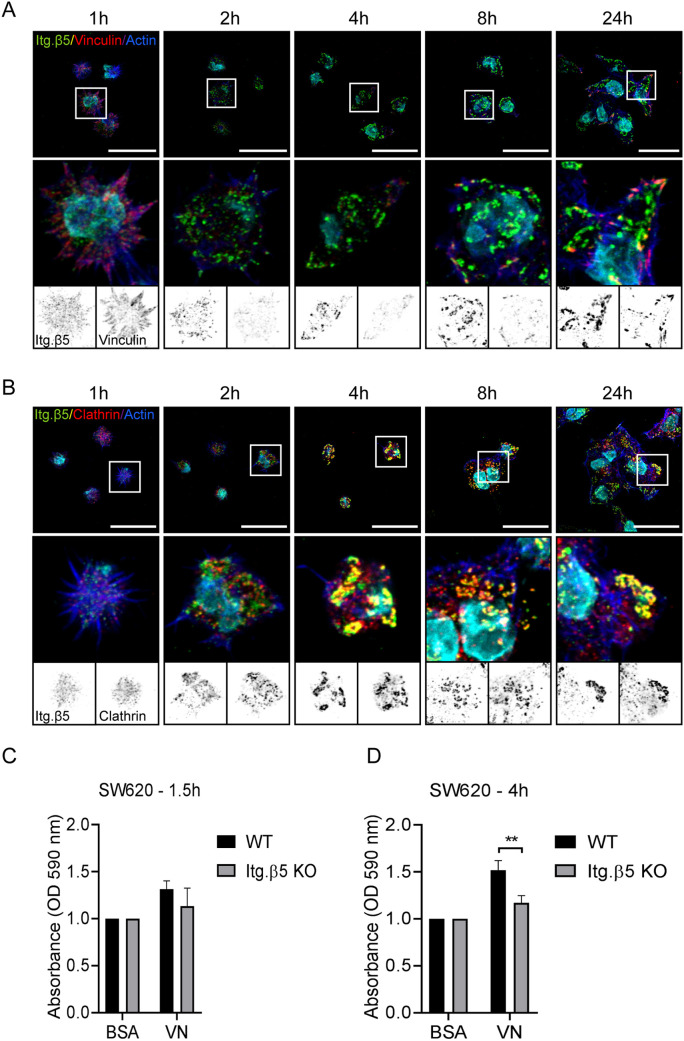
**Integrin β5 in clathrin lattices mediates adhesion to vitronectin ∼4 h after cell seeding.** (A,B) SW620 cells were seeded on vitronectin-coated coverslips and fixed at the indicated time points. Representative immunofluorescence images showing integrin β5 (green in merge), vinculin (A) or clathrin (B) (red in merge), actin in blue, and the cell nuclei in cyan. Scale bars: 20 μm. Representative images are shown of two independent experiments performed in duplicate. (C,D) Adhesion assay performed 1.5 h (C) and 4 h (D) after seeding SW620 wild-type (WT) and β5 knockout cells on vitronectin (VN) (three biological replicates; each experiment in triplicate; bars show mean±s.d). **P<0.01 (two-sided unpaired Student's *t*-test).

The main function of integrin β5 is to adhere to vitronectin. We wondered whether integrin β5 mediates adhesion to vitronectin differently in FAs versus FCLs. To address this, the assembly of β5-containing FCLs was analyzed by seeding SW620 cells on vitronectin-coated coverslips and staining cells 1, 2, 4, 8 and 24 h after cell seeding. We observed FA formation during early cell adhesion, although β5 at this time point showed a dispersed localization pattern (Fig. 7A). After 2–4 h, β5 formed larger structures that colocalized with clathrin molecules but not with vinculin, indicating the exclusive localization of β5 to FCLs (Fig. 7A,B). Of interest, cell spreading is associated with a decrease in Rho activity, which is in agreement with our findings that β5-containing FCLs are formed when cellular tension is low (Ren et al., 1999). Between 2–8 h after cell seeding, only small FAs could be detected in SW620 cells. After 24 h, both β5-containing FAs and FCLs could be observed, with SW620 cells favoring the formation of FCLs (Fig. 7A,B). To gain more insight into how integrin β5-containing FCLs play a role in cell–ECM adhesion, we compared integrin β5 wild-type and deficient SW620 cells in short (1.5 h) and long-term (4 h) adhesion assays. In the short-term adhesion assay, adhesion to vitronectin only showed a minor decrease in β5-deficient cells compared to wild-type control cells (Fig. 7C). This effect was increased when cells adhered to vitronectin for 4 h (Fig. 7D).

**Fig. 8.**
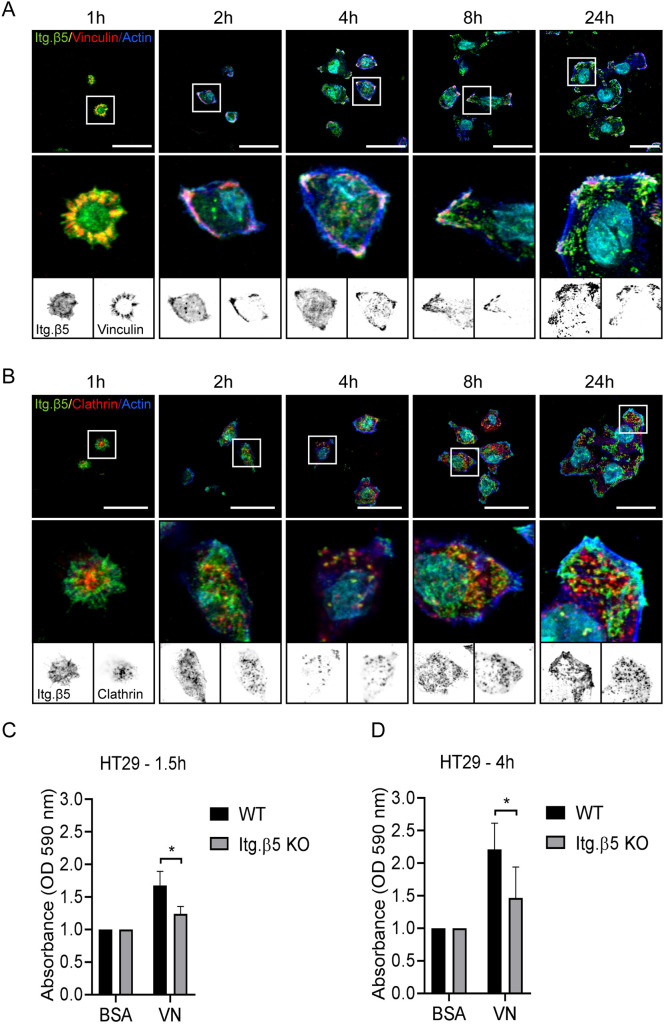
**Integrin β5 in focal adhesions mediates early adhesion to vitronectin.** (A,B) HT29 cells were seeded on vitronectin-coated coverslips and fixed at the indicated time points. Representative immunofluorescence images show integrin β5 (green in merge), vinculin (A) or clathrin (B) (red in merge), actin in blue, and the cell nuclei in cyan. Scale bars: 20 μm. Representative images are shown of two independent experiments performed in duplicates. (C,D) Adhesion assay performed 1.5 h (C) and 4 h (D) after seeding HT29 wild-type (WT) and β5 knockout cells on vitronectin (VN) (three biological replicates; each experiment in triplicate; bars show mean±s.d.). *P<0.05 (two-sided unpaired Student's *t*-test).

In contrast to SW620 cells, HT29 cells formed β5-containing FAs during early cell adhesion (Fig. 8A). Colocalization of β5 with clathrin structures could also be observed for the first time 2–4 h after cell seeding (Fig. 8B). Adhesion to vitronectin could be observed in both short- and long-term adhesion assays (Fig. 8C,D).

In conclusion, both β5-containing FAs and FCLs mediate adhesion to vitronectin, albeit with different dynamics.

## DISCUSSION

Integrin αVβ5 has a unique property – it can localize both in FAs and FCLs. Here, we studied the molecular mechanisms that control the subcellular distribution of integrin αVβ5 and investigated whether the function of β5 depends on its localization. Clathrin adaptor proteins, including ARH, Dab2 and Numb, are required for the formation of β5-containing FCLs and contain binding sites on the β5 cytoplasmic domain that overlap with that of talin (Lock et al., 2018; Zuidema et al., 2018; Calderwood et al., 2003). Our peptide pulldown and BioID experiments show that β5, but not β1 and β3, bind to Numb and ARH. In support of this, we were unable to detect binding of β1 and β3 to ARH and Numb by MST using the same concentration range at which binding of β5 could be detected. Additionally, the measurements show that the affinity of β5 for ARH (*K*_d_=5.6±1.4 µM; mean±s.d.) and Numb (*K*_d_=28.5±7.7 µM) is higher than that for the THD1 (*K*_d_=41.7±9.5 µM). However, THD1 bound with a slightly higher affinity to the β5 than to the β1 or β3 cytoplasmic domain.

It is important to mention that MST measurements to determine protein-binding affinities were performed with purified *in vitro* synthesized integrin β tails and recombinant THD1, whereas in living cells and total cell lysates used in BioID and peptide pulldown experiments, respectively, other proteins are present that can interact with full-length talin and stabilize its (proximity) interaction with the integrin β cytoplasmic domains. Of interest, talin and kindlin-1/2 cooperate to activate integrins by forming a ternary complex with the β cytoplasmic domain (Ma et al., 2008; Bledzka et al., 2012; Haydari et al., 2020; Theodosiou et al., 2016; Ye et al., 2013; Moser et al., 2008). Because kindlin-1/2 has a higher binding affinity for β1 than β5, we cannot exclude a (minor) role of kindlin-1/2 in determining the subcellular localization of β1 versus β5, as it might reinforce the interaction between β1 and talin in FAs. ARH is highly expressed in keratinocytes compared to the human cancer cell lines, where Dab2 and Numb might play a more prominent role in regulating the subcellular distribution of β5.

In contrast to β1, β3 and β5 did not bind kindlin-1/2 in the peptide pulldown experiments, but interacted strongly with SNX17. Because SNX17 and kindlin share the same binding site on the β1 cytoplasmic domain (Böttcher et al., 2012), it is possible that in the peptide pulldown experiments, which were carried out using whole cell lysates from RAC-11P cells, these proteins compete with each other for binding to the different cytoplasmic domain peptides, and that a higher affinity of β1 for kindlin-1/2 (Fig. S2) prevents the binding of β1 to SNX17 in the peptide pulldown experiments. However, this may only be pertinent when the amount of cytoplasmic domain peptides has been limited. Consistent with the results of the pulldown experiments, MST measurements revealed that β1 binds kindlin-2 with a much higher affinity than β3 and β5.

Taken together, the interactions between the integrin β subunits and their adaptor proteins might result in the distinct subcellular distribution patterns of the integrin receptors.

An additional level of regulation of the integrin β5 localization could be accomplished by phosphorylation of the SERS region. Site-directed mutagenesis of the β5 S759/S762 to phosphoserine-mimicking glutamate residues resulted in increased localization of β5 in FCLs. Previous studies reported that PAK4 binds β5-SERS and regulates its phosphorylation (Li et al., 2010; Zhang et al., 2002), although β5 was not identified in the PAK4 interactome (Zhao et al., 2017) and a recent study reports that PAK4 does not phosphorylate β5 (Alfonzo-Méndez et al., 2022). We identified PAK2, PAK3 and PAK6 proteins in proximity to the integrin subunits β3, β4 and β5 (Zuidema et al., 2018; Te Molder et al., 2020), but did not find an interaction between β5 and any of the PAK proteins. Instead, we identified the serine/threonine-protein kinase MARK2 as a β5-associated protein and demonstrated that MARK2 promotes clustering of wild-type β5 in FCLs, but not that of the β5-S759/762E mutant, which is already predominantly localized in FCLs. The finding that a disruptive S>A mutant of the SERS region did not impair the assembly of β5-containing FCLs and these adhesion structures can be formed independently of FAs (Zuidema et al., 2018; Lock et al., 2018) indicates that β5 does not need to be phosphorylated to reside in FCLs. Therefore, it would not be expected that β5 phosphorylation has a major role in its redistribution. In line with this, calyculin A treatment only led to a minor redistribution of the integrin. Unfortunately, efforts to demonstrate that MARK2 regulates β5 localization through its function as a kinase that phosphorylates β5 S759/762 have been unsuccessful – neither through mass spectrometry analysis of β5 immunoprecipitates prepared from calyculin A-treated cells, nor *in vitro* kinase assays performed with β5 immunoprecipitates (which has been shown to contain MARK2; Fig. 4C, data not shown) were we able to detect the phosphorylation of β5 at Ser-759/762.

In line with our previous findings (Wang et al., 2020; Zuidema et al., 2018), we observed that β5 clustering in FAs is favored in cells that express constitutively active RhoA and that the localization of β5 in different cancer cells is positively correlated with the amount of cellular tension. GEF-H1 is a microtubule-associated Rho-GEF that couples microtubule dynamics to cell contractility (Joo and Olson, 2021). GEF-H1 is inactive when bound to microtubules and becomes activated after being released from microtubules upon treatment with the microtubule-depolymerizing drug nocodazole (Krendel et al., 2002). The data that nocodazole inhibits the localization of β5 in FCLs and increased cell tension (Fig. 5E–G) suggests an important role of microtubule dynamics in determining the subcellular localization of β5. Previously, it was shown that MARK2 can phosphorylate GEF-H1 at S886 and that subsequent phosphorylation of this serine residue inhibits the activity of GEF-H1 by inducing its binding to microtubules (Yamahashi et al., 2011). However, knockdown of MARK2 in PA-JEB and PA-JEB/β4 keratinocytes did not decrease the phosphorylation of GEF-H1, but in fact increased its phosphorylation. A role of this protein in regulating the subcellular localization of β5 through phosphorylation of GEF-H1 at S886 therefore seems unlikely.

In addition to MARK2 and GEF-H1, we identified several other microtubule-associated proteins as β5-interacting proteins, including kinesin-1, EB1, HOOK2 and NUDC, and these proteins might play a role in regulating microtubule-based trafficking of β5. The impaired ability of integrin β5-S759/762E and -S759/762A mutants to response to nocodazole treatment (Fig. S4B–E) might be due to disrupted interactions of these microtubule-related proteins to the mutated residues.

To address whether the localization of β5 in FAs versus FCLs differentially regulates its function, we selected three colorectal cancer cell lines that contain β5 mainly in FAs (HT29), in FCLs (SW620) or in roughly equal levels in both adhesion complexes (SW480). All cell lines showed significantly reduced proliferation after inhibition or deletion of β5. It has been reported that depletion of β5 does not affect the G1, S, and G2 cell cycle phases (Bianchi-Smiraglia et al., 2013; Lock et al., 2018). Knockdown of β5 in breast cancer cells was accompanied by decreased FAK and ERK signaling (Bianchi-Smiraglia et al., 2013); while we also observed decreased levels of phosphorylated FAK, mainly in β5-deficient HT29 and U251MG, we did not detect obvious changes in ERK activity. Lock et al. observed β5 in retraction fibers that lack vinculin and proposed a role for β5 in ‘reticular adhesions’ during mitosis, which would offer dividing cells sites of ECM attachment when FAs disassemble (Lock et al., 2018). At the same time, other studies show that the integrin αV and β1 subunits also remain present at cell–ECM contact sites after disassembly of FAs during mitosis (Dix et al., 2018; Chen et al., 2022), indicating that reticular adhesions or FCLs do not exclusively mediate adhesion during mitosis. Based on our data, we conclude that β5, both in FAs and FCLs, promotes cell proliferation by mediating adhesion to vitronectin, as impaired cell proliferation caused by inhibition or deletion of β5 could be rescued by culturing β5-deficient cells on collagen-coated plates. Therefore, the impaired proliferation that we observed was caused by a general adhesion defect to vitronectin and was not achieved by one particular β5-containing adhesion complex. Both β5-containing FAs and FCLs mediate adhesion to vitronectin, although the complexes are formed with different dynamics – FAs are assembled earlier than FCLs and are therefore most likely more important in mediating early cell adhesion and spreading on vitronectin.

Based on our findings, we conclude that the amount of cellular tension regulates the subcellular distribution of β5. Interestingly, a recent study demonstrates that the formation of clathrin plaques is regulated by alternative splicing of exon 31 of the clathrin heavy chain gene (CLTC), resulting in the inclusion of a 7-amino-acid sequence within the trimerization domain of the clathrin heavy chain (Moulay et al., 2020). Cells that usually would lack clathrin plaques were able to form them after modulating the splicing of exon 31 (Moulay et al., 2020). These findings raise the question of whether certain cell types are genetically programmed to assemble FCLs. In addition, it would be of interest to study the first event in the formation of FCLs, which could be the frustrated endocytosis of integrins, as we and others have proposed (Zuidema et al., 2020; Baschieri et al., 2018; Zuidema et al., 2018). Alternatively, there could be a cell intrinsic mechanism that starts the formation of FCLs, to which cell surface receptors are subsequently recruited to stabilize these structures by providing anchoring to the ECM. Further investigation will be needed to address these questions.

In conclusion, integrin β5 promotes cancer cell proliferation by mediating adhesion to vitronectin. This role of β5 can be accomplished in FAs as well as in FCLs. Integrin β5 clustering in FCLs is promoted when cellular tension is low and is likely mediated by its interaction with ARH and phosphorylation of its SERS region. In contrast, high actomyosin contractility favors the assembly of β5-containing FAs.

## MATERIALS AND METHODS

### Reagents

Primary antibodies used are listed in Table S4. Secondary antibodies were as follows: goat anti-rabbit-IgG conjugated to Alexa Fluor 488, goat anti-mouse-IgG conjugated to Alexa Fluor 488, goat anti-mouse-IgG conjugated to Texas Red, goat anti-mouse-IgG conjugated to Alexa Fluor 568, donkey anti-rabbit-IgG conjugated to Alexa Fluor 594, goat anti-rabbit-IgG conjugated to Alexa Fluor 647, and goat anti-mouse-IgG conjugated to Alexa Fluor 647 (Invitrogen), PE-conjugated donkey anti-rabbit-IgG antibody (Biolegend #406421), stabilized HRP-conjugated goat anti-mouse-IgG and HRP-conjugated goat anti-rabbit-IgG (Bio-Rad). A conjugated streptavidin-HRP (1:1000; GE Healthcare #RPN1231) antibody was used for detection of biotinylated proteins. Calyculin A (#9902) was from Cell Signaling Technology and nocodazole from Sigma-Aldrich.

### Cell lines

Immortalized keratinocytes were isolated from a patient with pyloric atresia associated with junctional epidermolysis bullosa (PA-JEB), as published elsewhere (Schaapveld et al., 1998). The derivation of this cell line was done for diagnostic purposes, thus the research conducted using these cells was exempt of the requirement for ethical approval. PA-JEB/β4 keratinocytes stably expressing integrin β4 were generated by retroviral transduction, as described previously (Sterk et al., 2000). Cells were maintained in serum-free keratinocyte medium (KGM; Invitrogen) supplemented with 50 μg ml^−1^ bovine pituitary gland extract, 5 ng ml^−1^ EGF and antibiotics (100 units ml^−1^ streptomycin and 100 units ml^−1^ penicillin).

RAC-11P (Sonnenberg et al., 1993) and A549, HT29, SW480, SW620, and U251MG cell lines, obtained from the American Type Culture Collection, were cultured in Dulbecco's modified Eagle's medium (DMEM) containing 10% heat-inactivated FCS and 100 units ml^−1^ each of streptomycin and penicillin (Sigma).

MCF7 and MDA-MB-231 cell lines were obtained from the research group of Lodewyk F. A. Wessels (The Netherlands Cancer Institute, Div. of Molecular Carcinogenesis, Amsterdam) and authenticated by suppliers using short tandem repeat profiling (Jastrzebski et al., 2018). MCF7 cells are maintained in DMEM and MDA-MB-231 in RPMI medium, both supplemented with 10% heat-inactivated FCS and antibiotics. All cells were cultured at 37°C in a humidified, 5% CO_2_ atmosphere.

### Transient transfection

MARK2 (M-004260-02-0010) siGENOME SMARTpool siRNAs were purchased from Dharmacon. The cDNA encoding constitutively active [LZRS-IRES-GFP-RhoA(V14)] RhoA was kindly provided by Jacques Neefjes (Leiden University Medical Center, Dept. of Cell and Chemical Biology, The Netherlands). Cells were transiently transfected by using 20 μl ml^−1^ Lipofectamine^®^ 2000 (Invitrogen) and 6.5 μg ml^−1^ cDNA solutions in Opti-MEM. After mixing these solutions 1:1 and incubating for 30 min at room temperature, cells were incubated with the transfection solution overnight.

### Generation of integrin β5-deficient cell lines

The target sgRNA against *ITGB5* (exon 3; 5′-ACGGTCCATCACCTCTCGGT-3′) was cloned into pX330-U6-Chimeric_BB-CBh-hSpCas9 [Addgene plasmid #42230, deposited by Feng Zhang (Cong et al., 2013)]. HT29 and SW620 cells were transfected with this vector in combination with a blasticidin cassette, as previously described (Blomen et al., 2015). Integrin β5-deficient cells were selected by supplementing the culture medium with 4 μg ml^−1^ blasticidin (Sigma) for 4 days following transfection and a subsequent FACS sort of the β5-negative cell population.

### Stable cellular transduction

The generation of pcDNA3-β5-BirA*(R118G) vector, the expression vectors encoding β5^ex^/β1^in^ and β5^ex^/β3^in^ chimeric integrin subunits were described previously (Zuidema et al., 2018). Point mutants of β5 S759/S762 were generated by site-directed mutagenesis with the PCR-based overlap extension method using Pfu DNA polymerase (Promega) and were subcloned into the BstEII and XbaI sites of the pcDNA3-β5-BirA*(R118G) vector. The DNA fragment encoding the β5 cytoplasmic domain with the 12-amino-acid stretch of β3 (WDTANNPLYKEA) was ordered as a geneblock from Integrated DNA Technology (IDT) and subcloned into the existing HindIII sites. Retroviral vectors containing mutant β5 cDNAs were generated by subcloning the mutant β5 cDNAs into the EcoRI and XhoI restriction sites of the LZRS-MS-IRES-Zeo vector. PA-JEB/β4 keratinocytes expressing different β5 mutants were generated by retroviral transduction.

### Flow cytometry

Cells were treated as indicated, trypsinized, and washed in PBS containing 2% FCS, followed by primary antibody incubation for 45 min at 4°C. Then, cells were washed three times in PBS containing 2% FCS and incubated with PE-conjugated secondary antibody for 45 min at 4°C. Next, after subsequent washing steps, cells were analyzed on an Attune NxT (Thermo Fisher Scientific) flow cytometer. For FACS sorting, the desired cell populations were isolated using a Becton Dickinson FACSAria IIu or Beckman Coulter Moflo Astrios cell sorter.

### Immunofluorescence

PA-JEB/β4 keratinocytes were seeded on glass coverslips and cultured in complete KGM medium for 24 h, and then treated with DMEM plus 10% FCS for 24 h. Other cell lines were seeded on glass coverslips and cultured in DMEM plus 10% FCS. Cells were fixed with 2% paraformaldehyde (PFA) for 10 min, permeabilized with 0.2% Triton X-100 for 5 min, and blocked with PBS containing 2% bovine serum albumin (BSA; Sigma) for at least 30 min. Next, cells were incubated with the primary antibodies for 1 h at room temperature. Cells were washed three times before incubation with the secondary antibodies for 1 h. Additionally, nuclei were stained with DAPI, and filamentous actin was visualized using Alexa Fluor 488 or 647-conjugated phalloidin (Invitrogen). After three washing steps with PBS, the coverslips were mounted onto glass slides in Mowiol. Images were obtained using a Leica TCS SP5 confocal microscope with a 63× (NA 1.4) oil objective.

### BioID assay and western blotting

PA-JEB/β4 cells expressing β5-BirA*, β5^ex^/β1^in^-BirA*, or β5^ex^/β3^in^-BirA were grown on 100 mm plates in complete KGM for 24 h, followed by DMEM supplemented with 50 µM biotin (Sigma #B4501) for 24 h. Cells were washed in cold PBS, lysed in RIPA buffer (20 mM Tris-HCl pH 7.5, 100 mM NaCl, 4 mM, 1% NP-40, 0.1% SDS and 0.5% sodium deoxycholate) supplemented with a protease inhibitor cocktail (Sigma), and cleared by centrifugation at 14,000 ***g*** for 30 min at 4°C. Lysates were incubated with Streptavidin Sepharose High Performance beads (GE Healthcare) overnight at 4°C. Beads were washes three times with NP40 buffer (20 mM Tris-HCl pH 7.5, 100 mM NaCl, 4 mM EDTA pH 7.5 and 1% NP-40) and twice with PBS, and the isolated biotinylated proteins were eluted in sample buffer (50 mM Tris-HCl pH 6.8, 2% SDS, 10% glycerol, 12.5 mM EDTA and 0.02% Bromophenol Blue) containing a final concentration of 2% β-mercaptoethanol and denatured at 95°C for 10 min.

Proteins were separated by electrophoresis using a Bolt Novex 4–12% gradient Bis-Tris gels (Invitrogen) or 15% SDS-PAGE gels (made in-house), transferred to Immobilon-P transfer membranes (Millipore Corp) and blocked for at least 30 min in 2% BSA in TBST buffer (10 mM Tris-HCl pH 7.5, 150 mM NaCl and 0.3% Tween-20). Primary antibody (diluted 1:1000 in 2% BSA in TBST buffer) incubation took place overnight at 4°C. After washing twice with TBST and twice with TBS, blots were incubated for 1 h hour at room temperature with horseradish peroxidase-conjugated goat anti-mouse-IgG or goat anti-rabbit-IgG (diluted 1:3000 in 2% BSA in TBST buffer). After subsequent washing steps, the bound antibodies were detected by enhanced chemiluminescence using SuperSignal™ West Dura Extended Duration Substrate (ThermoFisher) or Clarity™ Western ECL Substrate (Bio-Rad) as described by the manufacturer. Signal intensities were quantified using ImageJ.

### Integrin tail peptide pulldowns

Peptide pulldowns were carried out as previously described (Böttcher et al., 2012) with the mouse β1 wild-type cytoplasmic tail peptide (HDRREFAKFEKEKMNAKWDTGENPIYKSAVTTVVNPKYEGK-OH) and a scrambled peptide (EYEFEPDKVDTGAKGTKMAKNEKKFRNYTVHNIWESRKVAP-OH), mouse β3 wild-type tail peptide (HDRKEFAKFEEERARAKWDTANNPLYKEATSTFTNITYRGT-OH), mouse β5 wild-type tail peptide (DRREFAKFQSERSRARYEMASNPLYRKPISTHTVDFAFNKFNKSYNGSVD-OH), β5 Δ8aa tail peptide (DRREFAKFQSERSRARYEMASNPLYRKPISTVVNKSYNGSVD-OH) and scrambled peptide RYGNAEYDPRRVKLSRFFENTNTFDHSSEKAMARKKSNFDFSVYQARISPH.

The tail peptides were synthesized *de novo* with a desthiobiotin on the N-terminus, coupled to Dynabeads MyOne streptavidin C1 (10 mgml^−1^, Invitrogen) and incubated with RAC-11P cell lysates (prepared with M-PER reagent, Thermo Fisher Scientific) at 4°C with rotation for 4 h. After three washes with wash buffer (M-PER diluted 1:1 with PBS), bound proteins were eluted in 2× Laemmli buffer at 95°C for 5 min and separated on an 8% SDS-PAGE gel.

### Recombinant protein production

#### Production of ARH, Numb-PTB, THD1 and kindlin-2 production for MST measurements

The full-length human ARH gene was cloned into the pETNKI-6xhis-3C-LIC vector (Luna-Vargas et al., 2011) and 6xhis–ARH protein was produced in Bl21(DE3) *E. coli* cells. Cells were grown at 37°C until an optical density at 600 nm (OD600) of 0.6 was reached. Temperature was reduced to 30°C and protein was expressed upon induction with 0.4 mM IPTG for 3.5 h. Cells were harvested and resuspended in lysis buffer (20 mM HEPES, pH 7.5, 200 mM NaCl, 1 mM TCEP and 5 μg ml^−1^ DNase). After sonication, cell debris and insoluble proteins were removed by centrifugation (21,000 ***g*** for 30 min). 6xhis–ARH was purified from the soluble fraction using nickel Sepharose beads and eluted in 20 mM HEPES, pH 7.5, 200 mM NaCl, 1 mM TCEP and 250 mM imidazole. Fractions containing 6xhi–ARH were further purified on a SEC650 size exclusion column (Bio-Rad), equilibrated with 20 mM HEPES, pH 7.5, 100 mM NaCl and 1 mM TCEP.

A pETNKI-6xhis-3C-Numb-PTB (20-175) construct was expressed in Rosetta2 (DE3) cells for 18 h at 20°C. Cells were lysed (25 mM Tris-HCl pH 8.0, 10 mM imidazole, 200 mM NaCl, 1 mM TCEP, 5 μg/ml DNase) and sonicated, after which insoluble proteins and cell debris were removed by centrifugation (21,000 ***g*** for 30 min at 4°C). Soluble lysate was applied to a nickel Sepharose column and beads were washed with wash buffer (lysis buffer without DNase) before protein was eluted in wash buffer supplemented with 250 mM imidazole. The 6xhis tag was cleaved off by his-3C protease during dialysis against 25 mM Tris-HCl pH 8.0, 100 mM NaCl, 1 mM TCEP, 16 h at 4°C. Elution fractions were pooled, diluted with six volumes of 20 mM HEPES pH 7.5, 1 mM TCEP and applied to a 1 ml Resource S cation-exchange column (Cytiva). Protein was eluted in a NaCl gradient (50–700 mM) in 25 mM HEPES (pH 7.5). 6xhis-Numb-PTB was concentrated and further purified by size-exclusion chromatography (SEC70 column, Bio-Rad) in 25 mM Tris-HCl pH 8.0, 200 mM NaCl and 1 mM TCEP.

pCoofy17-THD1 (1-405) constructs were transformed into Rosetta2(DE3) cells and his-SUMO-THD1 proteins were expressed for 16 h at 20°C after induction with 0.4 mm IPTG. Cells were harvested and resuspended in 25 mM Tris-HCl pH 8.0, 10 mM imidazole, 200 mM NaCl, 1 mM TCEP and 5 μg ml^−1^ DNase, and lysed by sonication. The soluble lysate fraction was collected after centrifugation (21,000 ***g*** for 30 min) and applied to nickel Sepharose beads. Beads were washed with 25 mM Tris-HCl pH 8.0, 200 mM NaCl, 1 mM TCEP and 10 mM imidazole, and proteins were eluted in the same buffer containing 250 mm imidazole. His-Senp2 protease was added to the proteins to remove the SUMO tag and the solutions were dialyzed against 25 mM Tris-HCl pH 8.0, 200 mM NaCl and 1 mM TCEP for 16 h at 4°C. After cleavage, solutions were passed over nickel beads to remove his-SUMO, residual uncleaved his-SUMO-THD1 and his-Senp2 protease. THD1 proteins were further purified by size exclusion chromatography on a S200 Superdex 16/60 column (Cytiva).

Kindlin-2 was expressed and purified as described in Böttcher et al. (2017). Briefly, kindlin constructs were cloned into pCoofy17 (Scholz et al., 2013), which adds an N-terminal His10-Sumo tag and expressed soluble in *E. coli* Rosetta at 18°C overnight. After purification by immobilized-metal affinity chromatography (IMAC) in high-salt TBS buffer (20 mM Tris, pH 7.5, 500 mM NaCl, 1 mM TCEP), the Sumo tag was removed by SenP2 (obtained from MPIB Biochemistry Core Facility) digest overnight and the protein was further purified by SEC using TBS (20 mM Tris-HCl pH 7.5, 200 mM NaCl, 1 mM TCEP) containing 5% glycerol as running and storage buffer. After the final chromatography, the purity, integrity and identity of recombinant kindlin-2 and talin-1 head domain were controlled by UV spectrum, SDS-PAGE, high-resolution total mass and dynamic light scattering (DLS).

### MST measurements

All MST measurements were performed on a Monolith NT.115 red-blue (Nanotemper, Munich, Germany) using premium-coated capillaries to reduce non-specific interaction of the proteins with the glass surface. Both interaction partners (ligand and receptor) were transferred into MST buffer (20 mM Tris-HCl pH 7.5, 200 mM sodium chloride, 1 mM TCEP and 0.05% Tween-20) to avoid artifacts derived from buffer mismatches. 50–200 nM Atto488-labeled integrin-β cytoplasmic tail (synthesized by the MPIB Core Facility) were used as ligands. The measurements were carried out at 10 to 20% LED power and 20 and 40% MST power. Data was analyzed using the MO Affinity Analysis Software (Nanotemper).

### Mass spectrometry

HaCat and PA-JEB/β4 keratinocytes were grown to 95% confluence in 15 cm dishes and lysed in 2 ml NP40 lysis buffer. After centrifugation for 60 min at 4°C cells, the supernatants were collected and incubated overnight at 4°C with Protein A Sepharose (GE Healthcare) coupled with antibodies. After washing three times with NP40 lysis buffer, and two times with PBS, bound protein were eluted by heating at 95°C in SDS-PAGE sample buffer and separated on a 4–12% SDS-PAGE gel. The gel was stained with Coomassie Blue, and lanes were excised and then reduced by treating with dithiothreitol and alkylated with iodoacetamide. After digestion with trypsin (mass spectrometry grade; Promega), peptide mixtures were extracted, measured and analyzed as previously described (Wang et al., 2020), with the following exceptions. Peptide mixtures (33% of total digest) were loaded directly onto the analytical column and analyzed by nano-liquid chromatography tandem mass spectrometry (nanoLC-MS/MS) on an Orbitrap Exploris 480 mass spectrometer equipped with a Proxeon nLC1200 system (Thermo Scientific). Solvent A was 0.1% formic acid in water and solvent B was 0.1% formic acid in 80% acetonitrile. Peptides were eluted from the analytical column at a constant flow of 250 nl/min in a 80-min gradient, containing a 64-min linear increase from 7% to 26% solvent B, followed by an 11-min wash at 90% solvent B.

Raw data were analyzed by MaxQuant (version 2.0.1.0; Cox et al., 2014) using standard settings for label-free quantitation (LFQ). MS/MS data were searched against the a Swissprot human database (20,397 entries, release 2021_04) complemented with a list of common contaminants and concatenated with the reversed version of all sequences. LFQ intensities were Log2-transformed in Perseus (version 1.6.14.0) (Tyanova et al., 2016), after which proteins were filtered for at least two out of three valid values in at least one sample group. Differentially expressed proteins were determined using an unpaired, two-tailed Student's *t*-test [thresholds false discovery rate (FDR) 0.05 and S0 0.1].

### Proliferation assay

Cells were seeded at a density of 5×10^4^ cells per well in a 96-well plate and treated with cilengitide or DMSO, as indicated in the figure legend. Then cells were fixed with 4% PFA at 1, 2, 3, 4 or 5 days after seeding. After fixation, cells were washed twice with H_2_O, stained with Crystal Violet for 30 min at room temperature and washed extensively with H_2_O. Cells were air-dried overnight and lysed in 2% SDS, after which the OD value of Crystal Violet at 490 nm was determined using an Epoch microplate reader equipped with Gen5 software. Assays were performed in triplicates and repeated twice.

### Adhesion assay

For adhesion assays, 96-well plates were coated with 5 µg ml^−1^ vitronectin (Sigma, #SRP3186) for 2 h at 37°C. Cells were trypsinized, resuspended in serum-free DMEM, and seeded at a density of 10^5^ cells per well and incubated for 1.5 or 4 or at 37°C. Nonadherent cells were washed away with PBS and the adherent cells were fixed with 4% PFA for 10 min at room temperature, washed twice with H_2_O, stained with Crystal Violet for 30 min at room temperature and washed extensively with H_2_O. Cells were air-dried overnight and lysed in 2% SDS, after which absorbance was measured at 590 nm on an Epoch microplate reader using Gen5 software. The values were normalized to the control group. Assays were performed in triplicates and repeated twice.

### Image analysis and statistical analysis

Image analysis was performed using Fiji (ImageJ) (Schindelin et al., 2012; Schneider et al., 2012). To quantify integrin clustering in FAs (based on vinculin staining) versus FCLs (clathrin staining), background was subtracted in both channels using a bilateral filter and the region of interest (ROI) was selected at the cell periphery. Colocalization of integrin clusters and FAs or FCLs was determined using the Image Calculator (command ‘multiply’) on both channels and calculating the area of overlapping clusters as a percentage of the total integrin cluster area per cell using the Analyze Particle function.

Mann–Whitney or two-sided unpaired Student's *t*-test (two-tailed *P* value) was performed using GraphPad Prism (version 7.0c). In figures, statistically significant values are shown as *P<0.05; **P<0.01; ***P<0.001; ****P<0.0001. Graphs were made in GraphPad Prism and display data in bars showing mean±s.d. or show all data points represented as box-and-whisker plots, in which the box extends the 25th to 75th percentiles, the middle line indicates the median, and whiskers go down to the smallest value and up to the largest.

## Supplementary Material

Supplementary information

Reviewer comments
