# Supplementary material for: Molecular determinants of αVβ5 localization in flat clathrin lattices – role of αVβ5 in cell adhesion and proliferation
Source: J Cell Sci. 2022 Jun 6;135(11):jcs259465. doi: 10.1242/jcs.259465 (PMC9234671; doi:10.1242/jcs.259465)
Supplement: Supplementary information [file joces-135-259465-s1.pdf]

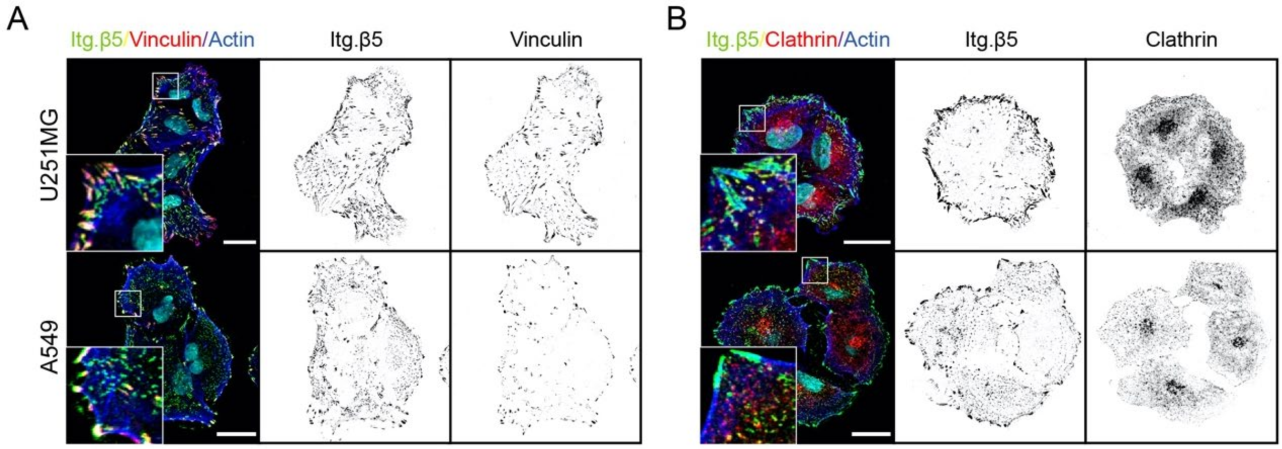

Fig. S1. (A,B) IF images of U251MG and A549 cells showing integrin β5 (green in merge), vinculin **(A)** or clathrin **(B)** (red in merge), actin (blue), DAPI (cyan). Scale bar, 20 µm.

Journal of Cell Science • Supplementary information

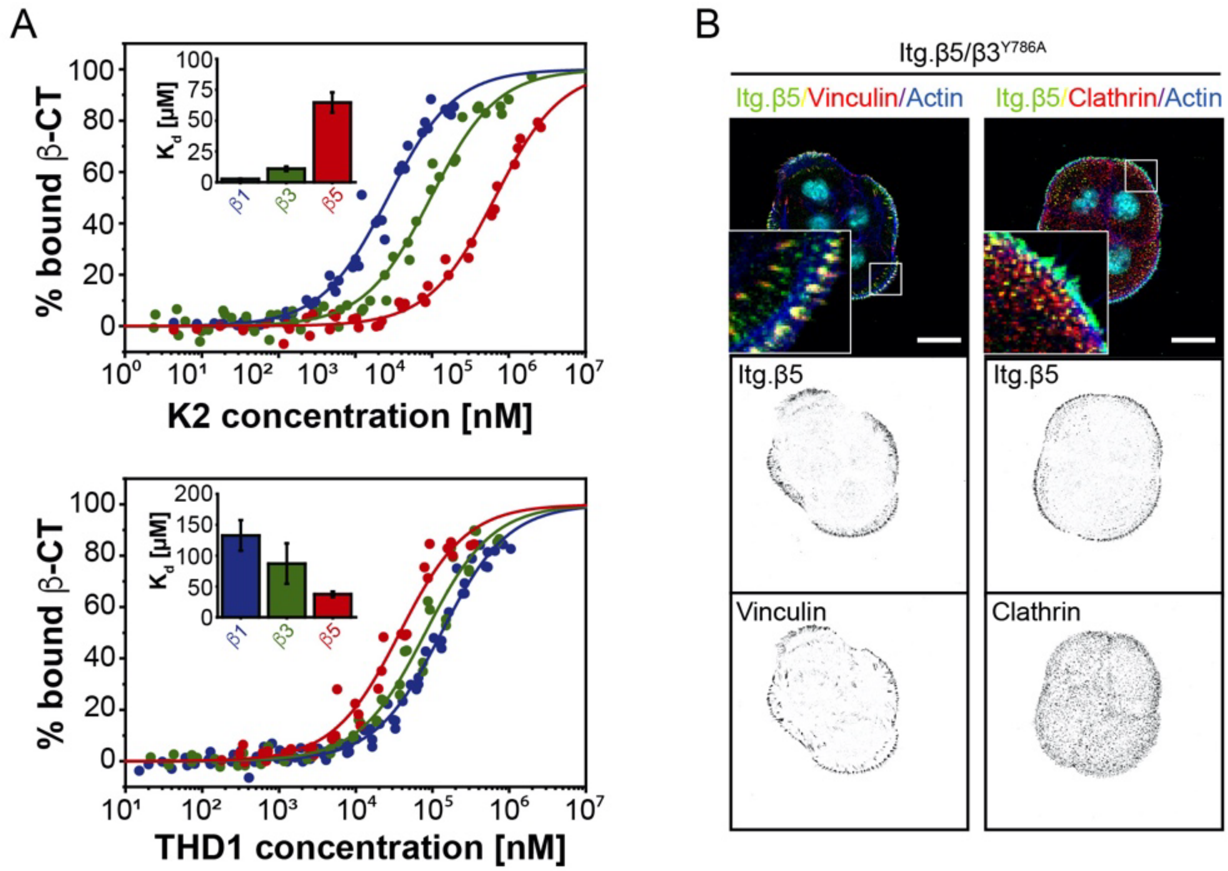

Fig. S2. (A) Titration of kindlin-2 (K2) and talin-1 head domain (THD1) to 200 nM ATTO488-labeled integrin β1, β3, and β5 cytoplasmic tail peptides (β-CT) to measure affinity using MST (n≥3). **(B)** Colocalization of the integrin $\beta5^{ex}/\beta3^{in}$ chimera containing a Y786A mutation in the MD-NxxY motif (green in merge) with vinculin (red; left panel) or clathrin (red; right panel) in β5-deficient PA-JEB/β4 keratinocytes. Actin is shown in blue. Nuclei are stained with DAPI (cyan). Scale bar, 20 µm.

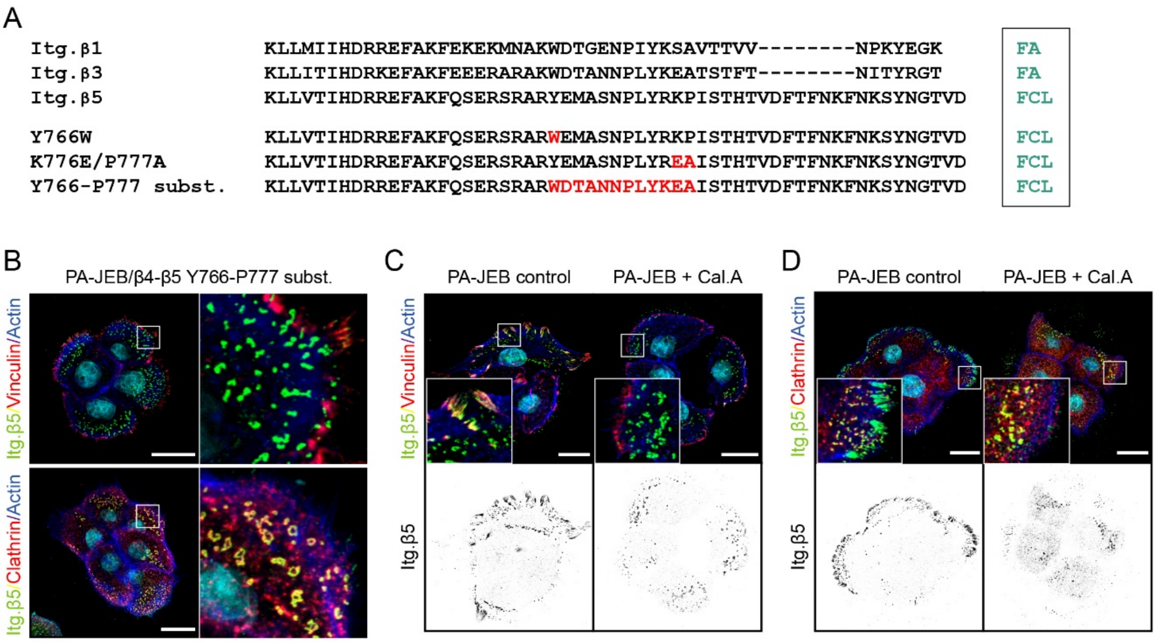

Fig. S3. **(A)** Amino acid sequences of the cytoplasmic domain of wild-type integrin β1, β3, β5 and β5 carrying mutations surrounding the membrane-proximal NPLY motif. The subcellular distribution of the integrin β subunits in FAs or FCLs is indicated. **(B)** Representative confocal microscopy images show that the integrin β5 mutant, carrying a substitution of 12 amino acids from β3 into β5 (Y766-P777 subst.) localizes predominantly in FCLs in PA-JEB/β4 keratinocytes **(C,D)** PA-JEB keratinocytes were grown in 10% FCS-supplemented DMEM culture medium overnight and then treated with 5 nM calyculin A (Cal.A) or DMSO (vehicle control) for 30 min prior to fixation. Merged images show integrin β5 (green), vinculin **(B,C)** or clathrin **(B,D)** (red), actin (blue) and the cell nuclei (cyan). Scale bar, 20 µm.

Journal of Cell Science • Supplementary information

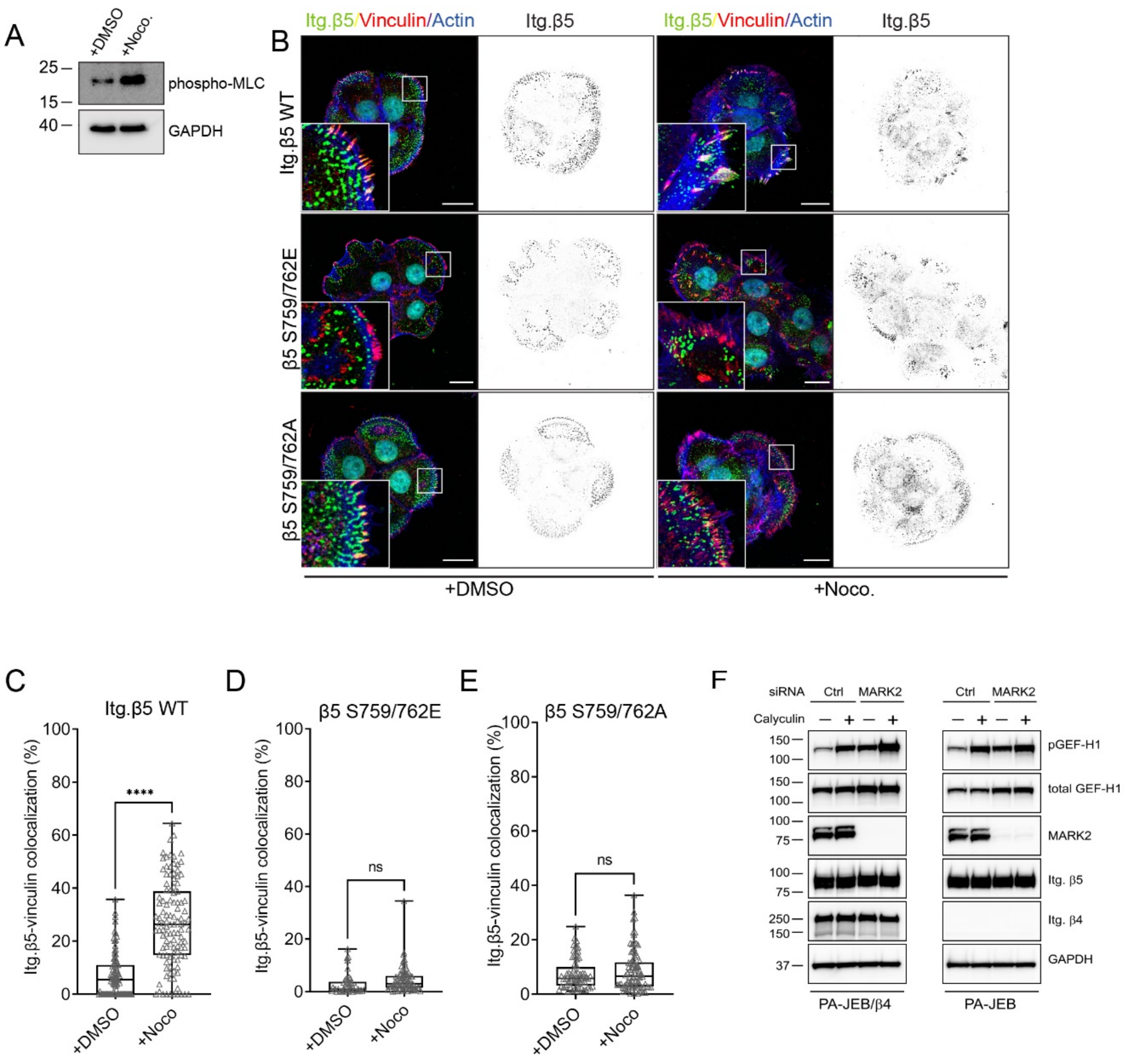

Fig. S4. (A) Representative western blots of phosphorylated myosin light chain (MLC) and GAPDH in PA-JEB/β4 keratinocytes treated with DMSO versus Nocodazole. (**B-E**) Analysis of integrin β5 clustering in FAs (**C**) in β5-deficient keratinocytes expressing wild-type β5, and β5 containing S759/762E or S759/762A mutations. Merged images show integrin β5 (green), vinculin (red), actin (blue) and the cell nuclei (cyan) (**C**). Scale bar, 20 µm. Quantifications of β5 or β5 mutants clustering in FAs are shown in (**C-E**). Data were obtained from three independent experiments. Total cells analyzed per condition: 90 (WT, DMSO), 111 (WT, Noco.) (**C**), 40 (S>E, DMSO), 80 (S>E, Noco.) (**D**), 65 (S>A, DMSO), 82 (S>A, Noco.) (**E**). Mann-Whitney U test was performed to determine statistical significance. ****, $P < 0.0001$. Box plots range from the 25th to 75th percentile; central line indicates the median; whiskers show smallest to largest value. **(F)** Representative western blots of phosphorylated GEF-H1, total GEF-H1 and MARK2 in PA-JEB/β4 and PA-JEB keratinocytes treated with or without siMARK2 combined with calyculin A. Integrin β4, β5 and GAPDH served as controls. (n=3).

Journal of Cell Science • Supplementary information

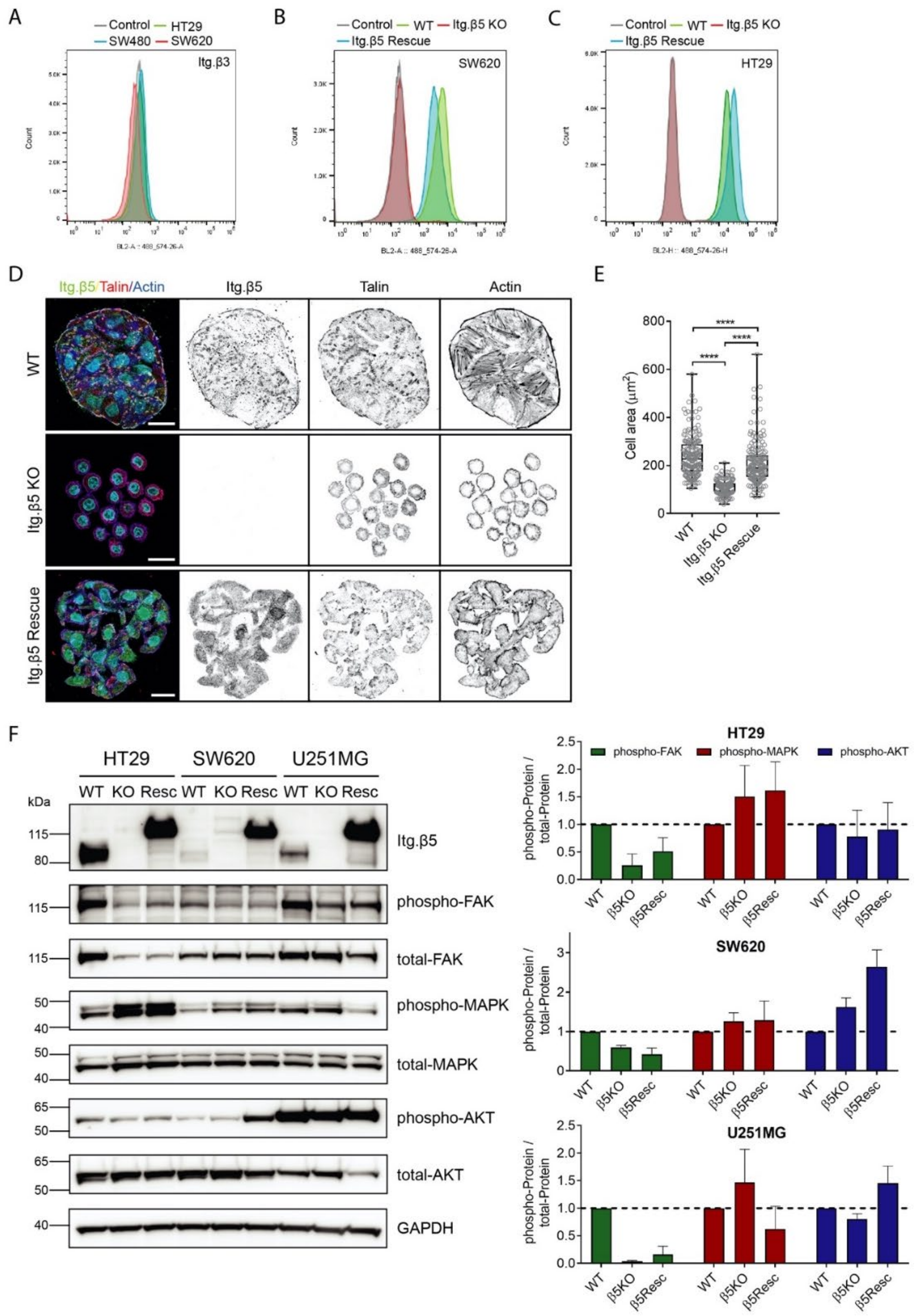

Fig. S5. (A) FACS plots showing the expression of integrin β3 in HT29, SW480, and SW620 cells. HT29 cells stained with a secondary PE-conjugated antibody only were used as negative control (n=3). **(B,C)** FACS plots showing the expression of β5 in SW620 **(B)** and HT29 **(C)** wild-type, β5 knockout and rescued cells. Cells stained with a secondary PE-conjugated antibody only were used as negative control (n=2-3). **(D)** HT29 cells were fixed after 5 days and merged images show integrin β5 (green), talin (red), actin (blue) and the cell nuclei (cyan). Scale bar, 20 µm. **(E)** Quantifications of the cell area of HT29 wild-type, β5 knockout and rescued cells. Data were obtained from three independent experiments. Total cells analyzed per condition: 179 (WT), 161 (KO), 172 (Resc.) Mann-Whitney U test was performed to determine statistical significance. ****, P < 0.0001. Box plots range from the 25th to 75th percentile; central line indicates the median; whiskers show smallest to largest value. **(F)** Representative western blots of phosphorylated FAK, MAPK, AKT in wild-type, β5-deficient (KO) and β5 rescued (Resc.) cell lines. Quantifications of signal intensities of the phosphorylated proteins normalized to total proteins levels are shown (n=3; bars show mean with s.d.).

Journal of Cell Science • Supplementary information

Table S1. Overview of cells that form integrin β5-containing FCLs

Cells	Adhesion complex	Reference
A375 human melanoma cells<br>A549 human lung carcinoma cells<br>U2OS human osteosarcoma cells<br>CS1 hamster melanoma cells<br>HeLa human cervical carcinoma cells<br>MCF7 human breast carcinoma cells<br>BT549 ductal breast carcinoma cells<br>Human hTERT microvascular endothelial (HME1) cells<br>Human hTERT immortalized retinal pigment epithelial (RPE1) cells<br>Primary mouse aortic endothelial (MAE) cells	"Reticular adhesions"	(Lock et al., 2018)
PA-JEB/β4 and HaCaT human keratinocytes	Flat clathrin lattices/plaques	(Zuidema et al., 2018)
HeLa human cervical carcinoma cells*<br>HepG2 human liver carcinoma*<br>Caco-2 human colorectal adenocarcinoma cells*<br>** When cultured on glass or on collagen-coated polyacrylamide 31 kPa gels*	"Clathrin-coated plaques"	(Baschieri et al., 2018)
HeLa human cervical carcinoma cells	Flat clathrin lattices/plaques	(Leyton-Puig et al., 2017)
Primary mouse myotubes	Flat clathrin lattices/plaques	(Vassilopoulos et al., 2014)
Primary rat myotubes	"Clathrin-coated membrane domains"	(De Deyne et al., 1998)
M21 human melanoma cells<br>H2981 and UCLA-P3 lung carcinoma cells	"Punctate distribution over the ventral cell surface outside FAs"	(Wayner et al., 1991)

Journal of Cell Science • Supplementary information

BASCHIERI, F., DAYOT, S., ELKHATIB, N., LY, N., CAPMANY, A., SCHAUER, K., BETZ, T., VIGNJEVIC, D. M., POINCLOUX, R. & MONTAGNAC, G. 2018. Frustrated endocytosis controls contractility-independent mechanotransduction at clathrin-coated structures. *Nat Commun,* 9**,** 3825.

DE DEYNE, P. G., O'NEILL, A., RESNECK, W. G., DMYTRENKO, G. M., PUMPLIN, D. W. & BLOCH, R. J. 1998. The vitronectin receptor associates with clathrin-coated membrane domains via the cytoplasmic domain of its beta5 subunit. *J Cell Sci,* 111 (Pt 18)**,** 2729-40.

LEYTON-PUIG, D., ISOGAI, T., ARGENZIO, E., VAN DEN BROEK, B., KLARENBEEK, J., JANSSEN, H., JALINK, K. & INNOCENTI, M. 2017. Flat clathrin lattices are dynamic actin-controlled hubs for clathrin-mediated endocytosis and signalling of specific receptors. *Nat Commun,* 8**,** 16068.

LOCK, J. G., JONES, M. C., ASKARI, J. A., GONG, X., ODDONE, A., OLOFSSON, H., GORANSSON, S., LAKADAMYALI, M., HUMPHRIES, M. J. & STROMBLAD, S. 2018. Reticular adhesions are a distinct class of cell-matrix adhesions that mediate attachment during mitosis. *Nat Cell Biol,* 20**,** 1290-1302.

VASSILOPOULOS, S., GENTIL, C., LAINE, J., BUCLEZ, P. O., FRANCK, A., FERRY, A., PRECIGOUT, G., ROTH, R., HEUSER, J. E., BRODSKY, F. M., GARCIA, L., BONNE, G., VOIT, T., PIETRI-ROUXEL, F. & BITOUN, M. 2014. Actin scaffolding by clathrin heavy chain is required for skeletal muscle sarcomere organization. *J Cell Biol,* 205**,** 377-93.

WAYNER, E. A., ORLANDO, R. A. & CHERESH, D. A. 1991. Integrins alpha v beta 3 and alpha v beta 5 contribute to cell attachment to vitronectin but differentially distribute on the cell surface. *J Cell Biol,* 113**,** 919-29.

ZUIDEMA, A., WANG, W., KREFT, M., TE MOLDER, L., HOEKMAN, L., BLEIJERVELD, O. B., NAHIDIAZAR, L., JANSSEN, H. & SONNENBERG, A. 2018. Mechanisms of integrin alphaVbeta5 clustering in flat clathrin lattices. *J Cell Sci,* 131.

Journal of Cell Science • Supplementary information

Table S2. Itg. β6 vs Itg. β5_IP_PA-JEB/β4

					Two color points Chart 1:	
Gene(s)	-LOG(P-value)	LFQ Abundance ratio (Itg.β6/Itg.β5) (2Log)	Significant or Not Significant FDR = 0.05 and S0 = 0.1	Significant or Not Significant_FDR = 0.05 and S0 = 0.1	Significant	Not significant
IGLV3-9	3,046	3,318	+	Significant	3,318	#N/A
ARHGAP10	4,541	3,607	+	Significant	3,607	#N/A
NOP56	3,240	3,635	+	Significant	3,635	#N/A
ANKRD28	2,092	-3,244	+	Significant	-3,244	#N/A
P4HA2	4,235	3,646	+	Significant	3,646	#N/A
KLK10	1,572	-1,717	+	Significant	-1,717	#N/A
HNRNPR	1,776	1,696	+	Significant	1,696	#N/A
HTRA2	3,736	-4,106	+	Significant	-4,106	#N/A
BUB3	2,754	1,813	+	Significant	1,813	#N/A
MYO1B	4,054	3,411	+	Significant	3,411	#N/A
PRMT3	1,566	-1,977	+	Significant	-1,977	#N/A
C;HIST1H2BD;HIST1H2	4,601	4,542	+	Significant	4,542	#N/A
DKC1	1,495	1,724	+	Significant	1,724	#N/A
H2AFY	3,061	2,289	+	Significant	2,289	#N/A
ECI2	2,425	2,079	+	Significant	2,079	#N/A
UBR5	2,962	-2,597	+	Significant	-2,597	#N/A
NDUFB4	2,331	2,092	+	Significant	2,092	#N/A
ASMTL	1,788	2,473	+	Significant	2,473	#N/A
STAU1	2,392	-3,848	+	Significant	-3,848	#N/A
AIFM1	3,776	2,890	+	Significant	2,890	#N/A
PLG	3,507	3,019	+	Significant	3,019	#N/A
ASS1	2,614	2,433	+	Significant	2,433	#N/A
C5	1,979	-1,223	+	Significant	-1,223	#N/A
LMNA	1,995	1,656	+	Significant	1,656	#N/A
TFRC	3,860	1,440	+	Significant	1,440	#N/A
RPLP0;RPLP0P6	2,117	-0,830	+	Significant	-0,830	#N/A
KRT18	2,723	0,958	+	Significant	0,958	#N/A
IGKV4-1	3,093	1,443	+	Significant	1,443	#N/A
ITGAV	4,376	-1,840	+	Significant	-1,840	#N/A
PCL1;HNRNPCL3;HNRN	1,966	2,276	+	Significant	2,276	#N/A
RHOC;RHOA	3,723	2,276	+	Significant	2,276	#N/A
HSP90AB1	2,916	-0,890	+	Significant	-0,890	#N/A
PDHA1	1,438	-2,069	+	Significant	-2,069	#N/A
LGALS1	4,904	3,580	+	Significant	3,580	#N/A
DLD	1,692	-1,450	+	Significant	-1,450	#N/A
H2AFV;H2AFZ	1,461	2,230	+	Significant	2,230	#N/A
FJ;HIST1H2AH;HIST1H2	2,694	1,792	+	Significant	1,792	#N/A
PABPC1	3,001	0,438	+	Significant	0,438	#N/A
HARS	2,417	1,934	+	Significant	1,934	#N/A
CKMT1A	2,446	-1,097	+	Significant	-1,097	#N/A
P4HA1	2,344	-2,646	+	Significant	-2,646	#N/A

Journal of Cell Science • Supplementary information

HNRNPL	1,741	1,978	+	Significant	1,978	#N/A
EZR	1,803	-0,968	+	Significant	-0,968	#N/A
HIST1H1B	1,369	2,592	+	Significant	2,592	#N/A
T1H1C;HIST1H1E;HIST1	3,791	3,614	+	Significant	3,614	#N/A
PRKACA;PRKACB	2,163	-2,032	+	Significant	-2,032	#N/A
PTPN1	2,271	2,104	+	Significant	2,104	#N/A
ITGB5	3,797	-9,138	+	Significant	-9,138	#N/A
RPL7	2,075	-0,537	+	Significant	-0,537	#N/A
ITGB6	4,613	7,066	+	Significant	7,066	#N/A
ITIH2	3,321	-4,168	+	Significant	-4,168	#N/A
LMNB1	1,502	2,049	+	Significant	2,049	#N/A
SDHB	4,055	-3,434	+	Significant	-3,434	#N/A
TGM2	1,317	-2,680	+	Significant	-2,680	#N/A
FBL	2,017	3,016	+	Significant	3,016	#N/A
HNRNPA2B1	1,781	1,862	+	Significant	1,862	#N/A
SFPQ	1,672	1,161	+	Significant	1,161	#N/A
CFL1	2,713	2,693	+	Significant	2,693	#N/A
DGKA	1,816	-1,688	+	Significant	-1,688	#N/A
AZGP1	3,060	-0,609	+	Significant	-0,609	#N/A
DDX6	2,691	-1,391	+	Significant	-1,391	#N/A
RPL13	2,011	-0,740	+	Significant	-0,740	#N/A
SDHA	3,755	-6,512	+	Significant	-6,512	#N/A
RRM2	4,351	-5,297	+	Significant	-5,297	#N/A
HNRNPH3	1,701	1,231	+	Significant	1,231	#N/A
HNRNPH1	3,266	4,403	+	Significant	4,403	#N/A
YWHAB	3,485	-1,871	+	Significant	-1,871	#N/A
PYCR1	3,905	-2,810	+	Significant	-2,810	#N/A
KIF5B	3,016	-2,852	+	Significant	-2,852	#N/A
MYH10	3,368	4,055	+	Significant	4,055	#N/A
ADD1	2,321	-2,061	+	Significant	-2,061	#N/A
DLST	3,790	-1,732	+	Significant	-1,732	#N/A
ATP6V1A	1,321	5,315	+	Significant	5,315	#N/A
EIF4A3	1,693	1,868	+	Significant	1,868	#N/A
RFC3	2,561	-1,602	+	Significant	-1,602	#N/A
TMPO	1,645	1,498	+	Significant	1,498	#N/A
RPS27	1,932	0,793	+	Significant	0,793	#N/A
MATR3	1,718	3,197	+	Significant	3,197	#N/A
CSNK1A1;CSNK1A1L	2,150	-0,964	+	Significant	-0,964	#N/A
CSNK1D	1,385	-3,044	+	Significant	-3,044	#N/A
NUMB	2,408	2,110	+	Significant	2,110	#N/A
HSD17B4	2,051	0,941	+	Significant	0,941	#N/A
HMGA2	3,275	1,428	+	Significant	1,428	#N/A
ACLY	4,584	-6,383	+	Significant	-6,383	#N/A
VCP	3,146	-4,291	+	Significant	-4,291	#N/A
NHP2L1	1,938	2,498	+	Significant	2,498	#N/A
HNRNPK	1,935	1,262	+	Significant	1,262	#N/A
YWHAG	4,423	-1,788	+	Significant	-1,788	#N/A
RPS8	2,212	-0,660	+	Significant	-0,660	#N/A
RPS15A	3,084	-0,355	+	Significant	-0,355	#N/A
YWHAE	5,886	-1,540	+	Significant	-1,540	#N/A

SNRPD3	2,590	1,957	+	Significant	1,957	#N/A
HIST1H4A	1,726	2,102	+	Significant	2,102	#N/A
RPL8	2,982	-0,930	+	Significant	-0,930	#N/A
YWHAZ	5,099	-1,661	+	Significant	-1,661	#N/A
GTF2I	2,948	-3,183	+	Significant	-3,183	#N/A
SLC25A11	2,523	0,509	+	Significant	0,509	#N/A
REL	2,530	1,645	+	Significant	1,645	#N/A
YWHAH	3,560	-2,246	+	Significant	-2,246	#N/A
CSTF1	2,848	2,495	+	Significant	2,495	#N/A
CALD1	1,551	-2,618	+	Significant	-2,618	#N/A
CKAP4	2,151	-0,809	+	Significant	-0,809	#N/A
KLC1	2,532	-3,055	+	Significant	-3,055	#N/A
GOLGA2	2,251	-2,818	+	Significant	-2,818	#N/A
ILF3	2,106	1,180	+	Significant	1,180	#N/A
CSTF3	2,970	1,971	+	Significant	1,971	#N/A
TRIM28	2,767	1,426	+	Significant	1,426	#N/A
G3BP1	3,934	2,077	+	Significant	2,077	#N/A
PKP1	1,681	1,144	+	Significant	1,144	#N/A
DDX39B;DDX39A	1,526	1,739	+	Significant	1,739	#N/A
CUX1	3,241	-3,089	+	Significant	-3,089	#N/A
CAPRIN1	4,080	4,006	+	Significant	4,006	#N/A
DSC3	2,179	2,226	+	Significant	2,226	#N/A
KPNB1	1,973	0,742	+	Significant	0,742	#N/A
NUMA1	2,245	2,056	+	Significant	2,056	#N/A
GAPVD1	2,583	-10,265	+	Significant	-10,265	#N/A
EBP	2,240	1,856	+	Significant	1,856	#N/A
PLCB4	3,696	-2,618	+	Significant	-2,618	#N/A
PLEC	2,111	0,872	+	Significant	0,872	#N/A
PCBP1	4,077	2,000	+	Significant	2,000	#N/A
SF3B3	2,386	2,005	+	Significant	2,005	#N/A
MAPRE1	2,268	-2,875	+	Significant	-2,875	#N/A
DPYSL2	1,431	1,757	+	Significant	1,757	#N/A
SRSF7	1,485	1,756	+	Significant	1,756	#N/A
UPP1	1,482	3,322	+	Significant	3,322	#N/A
UGP2	2,568	-1,294	+	Significant	-1,294	#N/A
IMMT	1,962	0,824	+	Significant	0,824	#N/A
SMU1	1,879	2,483	+	Significant	2,483	#N/A
TMEM201	1,770	0,806	+	Significant	0,806	#N/A
FAM160B1	2,287	-3,294	+	Significant	-3,294	#N/A
CDC42BPG	3,630	-3,465	+	Significant	-3,465	#N/A
PTRF	2,025	0,649	+	Significant	0,649	#N/A
MARK2	4,950	-8,079	+	Significant	-8,079	#N/A
PM20D2	2,761	2,858	+	Significant	2,858	#N/A
KIAA0319L	3,313	3,185	+	Significant	3,185	#N/A
LSM14A	3,584	-3,960	+	Significant	-3,960	#N/A
MCU	2,069	0,579	+	Significant	0,579	#N/A
NPLOC4	3,408	-4,260	+	Significant	-4,260	#N/A
GEMIN5	3,102	2,212	+	Significant	2,212	#N/A
TTN	4,298	-2,957	+	Significant	-2,957	#N/A
DDX1	3,071	0,786	+	Significant	0,786	#N/A

ARHGEF1	3,709	-4,986	+	Significant	-4,986	#N/A
ARHGEF2	3,429	-4,971	+	Significant	-4,971	#N/A
HOOK2	2,979	-3,713	+	Significant	-3,713	#N/A
EDC3	3,578	3,415	+	Significant	3,415	#N/A
GRAMD3	2,869	2,056	+	Significant	2,056	#N/A
DOCK7	3,216	0,893	+	Significant	0,893	#N/A
CLCC1	1,703	1,127	+	Significant	1,127	#N/A
HNRNPAB	1,388	1,791	+	Significant	1,791	#N/A
C9orf156	3,619	-3,401	+	Significant	-3,401	#N/A
PRKD2	1,978	1,882	+	Significant	1,882	#N/A
VPS33B	2,982	-2,284	+	Significant	-2,284	#N/A
C2orf44	3,465	-5,314	+	Significant	-5,314	#N/A
RPRD1B	2,819	2,038	+	Significant	2,038	#N/A
DDX21	1,903	2,703	+	Significant	2,703	#N/A
ABHD10	2,664	-1,901	+	Significant	-1,901	#N/A
TECR	2,270	0,548	+	Significant	0,548	#N/A
LIMA1	3,399	4,530	+	Significant	4,530	#N/A
PSME2	2,114	-2,077	+	Significant	-2,077	#N/A
CORO1C	2,268	1,837	+	Significant	1,837	#N/A
G3BP2	2,465	1,851	+	Significant	1,851	#N/A
NUDC	5,287	-9,348	+	Significant	-9,348	#N/A
NOP58	2,673	2,825	+	Significant	2,825	#N/A
SNX24	3,260	-3,295	+	Significant	-3,295	#N/A
MTCL1	6,898	-8,439	+	Significant	-8,439	#N/A
MYO5A	2,622	4,691	+	Significant	4,691	#N/A
SAMM50	3,107	2,143	+	Significant	2,143	#N/A
ARFGEF1	4,747	2,728	+	Significant	2,728	#N/A

Journal of Cell Science • Supplementary information

Table S3. Itg. β6 vs Itg. β5_IP_HaCat

					Two color points Chart 1:	
Gene(s)	-LOG(P-value)	LFQ Abundance ratio (ITGB6/ITGB5) (2Log)	Significant or Not Significant FDR = 0.05 and S0 = 0.1	Significant or Not Significant_FDR = 0.05 and S0 = 0.1	Significant	Not significant
IGLV3-9	3,647	4,764	+	Significant	4,764	#N/A
CUX2	4,866	-6,418	+	Significant	-6,418	#N/A
P4HA2	2,776	3,298	+	Significant	3,298	#N/A
HTRA2	2,725	-3,825	+	Significant	-3,825	#N/A
NUDT21	1,612	-1,364	+	Significant	-1,364	#N/A
PPL	2,140	1,788	+	Significant	1,788	#N/A
SYNCRIP	2,151	0,549	+	Significant	0,549	#N/A
PRMT3	3,245	-2,878	+	Significant	-2,878	#N/A
SF3B1	2,063	1,375	+	Significant	1,375	#N/A
ASMTL	2,587	1,611	+	Significant	1,611	#N/A
STAU1	4,005	-5,635	+	Significant	-5,635	#N/A
AIFM1	3,486	3,759	+	Significant	3,759	#N/A
ASS1	3,424	4,328	+	Significant	4,328	#N/A
A2M	3,241	0,844	+	Significant	0,844	#N/A
C3	2,481	0,963	+	Significant	0,963	#N/A
FN1	3,516	-0,897	+	Significant	-0,897	#N/A
TFRC	4,251	1,636	+	Significant	1,636	#N/A
TF	3,080	1,450	+	Significant	1,450	#N/A
RPLP0;RPLP0P6	3,072	-0,905	+	Significant	-0,905	#N/A
ITGAV	4,656	-2,505	+	Significant	-2,505	#N/A
P4HB	3,093	1,251	+	Significant	1,251	#N/A
PABPC1	2,982	1,116	+	Significant	1,116	#N/A
CKMT1A	2,711	-4,101	+	Significant	-4,101	#N/A
PTPN1	3,407	2,621	+	Significant	2,621	#N/A
ITGB5	3,398	-9,331	+	Significant	-9,331	#N/A
RPL7	2,049	-0,729	+	Significant	-0,729	#N/A
ITGB6	5,837	6,534	+	Significant	6,534	#N/A
NCL	3,249	-0,718	+	Significant	-0,718	#N/A
TRIM21	2,786	3,347	+	Significant	3,347	#N/A
ITIH2	4,624	-3,488	+	Significant	-3,488	#N/A
SDHB	2,445	-3,240	+	Significant	-3,240	#N/A
TGM2	1,627	-3,883	+	Significant	-3,883	#N/A
TUBG1;TUBG2	2,148	-1,217	+	Significant	-1,217	#N/A
DNAJB1	2,811	0,978	+	Significant	0,978	#N/A
ATP5A1	2,883	0,715	+	Significant	0,715	#N/A
DDX6	4,457	-7,882	+	Significant	-7,882	#N/A
PTBP1	3,813	3,390	+	Significant	3,390	#N/A
CALR	2,858	-2,595	+	Significant	-2,595	#N/A
CANX	3,278	-2,459	+	Significant	-2,459	#N/A
NOS1	3,207	2,764	+	Significant	2,764	#N/A

RPL12	1,894	-0,889	+	Significant	-0,889	#N/A
ADSL	2,261	-2,230	+	Significant	-2,230	#N/A
SDHA	2,964	-3,699	+	Significant	-3,699	#N/A
RRM2	4,475	-5,004	+	Significant	-5,004	#N/A
YWHAB	2,540	-0,679	+	Significant	-0,679	#N/A
RPL9	2,829	-0,527	+	Significant	-0,527	#N/A
KIF5B	2,756	-2,403	+	Significant	-2,403	#N/A
ADD1	1,545	-1,502	+	Significant	-1,502	#N/A
IGFALS	2,157	-1,410	+	Significant	-1,410	#N/A
ATP6V1A	5,813	7,405	+	Significant	7,405	#N/A
RPL3	2,487	-0,498	+	Significant	-0,498	#N/A
DDOST	2,707	-2,176	+	Significant	-2,176	#N/A
RPL5	2,564	-0,650	+	Significant	-0,650	#N/A
RPL28	2,722	-0,841	+	Significant	-0,841	#N/A
SERPINH1	2,548	1,259	+	Significant	1,259	#N/A
RPL14	3,829	-4,129	+	Significant	-4,129	#N/A
FXR1	3,339	-1,268	+	Significant	-1,268	#N/A
ACLY	3,691	-4,483	+	Significant	-4,483	#N/A
RPL15	4,164	-0,918	+	Significant	-0,918	#N/A
RPL27	2,066	-0,778	+	Significant	-0,778	#N/A
YWHAG	3,938	-1,235	+	Significant	-1,235	#N/A
YWHAE	3,451	-1,530	+	Significant	-1,530	#N/A
RPL30	2,562	-0,968	+	Significant	-0,968	#N/A
RPL10A	1,997	-0,693	+	Significant	-0,693	#N/A
YWHAZ	2,633	-1,092	+	Significant	-1,092	#N/A
TUBA4A	2,270	0,537	+	Significant	0,537	#N/A
GTF2I	2,530	-4,431	+	Significant	-4,431	#N/A
RPL19	2,078	-0,793	+	Significant	-0,793	#N/A
SORD	4,183	-2,960	+	Significant	-2,960	#N/A
KIF23	2,064	1,862	+	Significant	1,862	#N/A
REL	2,462	1,902	+	Significant	1,902	#N/A
YWHAH	2,915	-1,571	+	Significant	-1,571	#N/A
RPL18	1,517	-2,499	+	Significant	-2,499	#N/A
KLC1	2,592	-3,367	+	Significant	-3,367	#N/A
GOLGA2	3,272	-2,922	+	Significant	-2,922	#N/A
ILF2	1,405	-2,024	+	Significant	-2,024	#N/A
TRIM28	2,652	1,496	+	Significant	1,496	#N/A
G3BP1	4,639	3,225	+	Significant	3,225	#N/A
SQSTM1	2,384	1,439	+	Significant	1,439	#N/A
CUX1	3,793	-2,870	+	Significant	-2,870	#N/A
TRIM29	2,865	0,394	+	Significant	0,394	#N/A
DYNC1H1	4,855	-2,966	+	Significant	-2,966	#N/A
MAP7	3,541	-0,449	+	Significant	-0,449	#N/A
CAPRIN1	2,512	3,999	+	Significant	3,999	#N/A
GAPVD1	2,591	-7,647	+	Significant	-7,647	#N/A
PON3	4,823	-4,598	+	Significant	-4,598	#N/A
MAPRE1	3,665	-3,458	+	Significant	-3,458	#N/A
UPP1	1,584	2,921	+	Significant	2,921	#N/A
PTRF	3,381	0,715	+	Significant	0,715	#N/A
MTHFD1L	2,064	-0,631	+	Significant	-0,631	#N/A

MARK2	3,861	-8,390	+	Significant	-8,390	#N/A
LSM14A	2,896	-4,340	+	Significant	-4,340	#N/A
NPLOC4	2,320	-2,413	+	Significant	-2,413	#N/A
GEMIN5	1,548	1,578	+	Significant	1,578	#N/A
PPP1R13L	2,943	0,491	+	Significant	0,491	#N/A
TTN	4,414	-3,141	+	Significant	-3,141	#N/A
ARHGEF1	3,884	-5,010	+	Significant	-5,010	#N/A
UFD1L	2,658	-2,335	+	Significant	-2,335	#N/A
ARHGEF2	4,061	-5,655	+	Significant	-5,655	#N/A
HOOK2	3,353	-4,099	+	Significant	-4,099	#N/A
EDC3	5,349	4,711	+	Significant	4,711	#N/A
DOCK7	3,287	1,886	+	Significant	1,886	#N/A
C9orf156	2,575	-3,518	+	Significant	-3,518	#N/A
PRKD2	3,616	2,962	+	Significant	2,962	#N/A
VPS33B	2,304	-2,709	+	Significant	-2,709	#N/A
OSBPL3	2,268	0,608	+	Significant	0,608	#N/A
RABEP2	3,605	2,175	+	Significant	2,175	#N/A
C2orf44	2,433	-4,974	+	Significant	-4,974	#N/A
VIPAS39	2,249	-3,009	+	Significant	-3,009	#N/A
RPRD1B	2,213	2,055	+	Significant	2,055	#N/A
44448	2,102	1,946	+	Significant	1,946	#N/A
PSME2	4,357	-2,666	+	Significant	-2,666	#N/A
G3BP2	4,495	2,869	+	Significant	2,869	#N/A
NUDC	2,912	-9,358	+	Significant	-9,358	#N/A
SNX24	3,571	-3,336	+	Significant	-3,336	#N/A
MYO5A	2,559	3,808	+	Significant	3,808	#N/A
DNAJC15	2,506	-1,900	+	Significant	-1,900	#N/A
PEX16	1,949	-0,679	+	Significant	-0,679	#N/A
ARFGEF1	2,562	3,544	+	Significant	3,544	#N/A

Journal of Cell Science • Supplementary information

Table S4. Primary antibody list

Antibody	Clone	Obtained from	Host	Application
**Integrin β5**	EM09902	Simon Goodman (Merck KGaA)	Rabbit mAb	IF/FACS: 1:200
**Integrin β5 - cyto**	5HK2	Homemade	Rabbit pAb	WB: 1:1000<br>IP: 1 µl / sample
**Integrin β1 - cyto**	U19	Ulrike Mayer	Rabbit pAb	WB: 1:1000
**Integrin β3**	C17	Ellen van der Schoot	Mouse mAb	FACS: 1:100
**Integrin β6 - cyto**	5HK1	Homemade	Rabbit pAb	IP: 1 µl / sample
**Vinculin**	VIIF9	Marina Glukhova	Mouse mAb	IF: 1:5
**Talin**	8d4	Sigma	Mouse mAb	IF: 1:200<br>WB: 1:1000
**ARH/LDLRAP1**		AntibodyPlus (#A7093)	Rabbit pAb	WB: 1:1000
**Numb**	S.925.4	Invitrogen (#MA5-14897)	Rabbit mAb	WB: 1:1000
**Clathrin, Heavy chain**	X22	Thermo Fisher (#MA1-065)	Mouse mAb	IF: 1:400
**Dab2**		Cell Signaling (#12906)	Rabbit mAb	WB: 1:500
**Kindlin-1**		Homemade (Ussar et al., 2006)	Rabbit pAb	WB: 1:5000
**Kindlin-2**	3A3	EMD Millipore (#MAB2617)	Mouse mAb	WB: 1:1000
**KANK-2**		Sigma (#HPA015643)	Rabbit pAb	WB: 1:2000
**SNX17**		ProteinTech (#10275-1-AP)	Rabbit pAb	WB: 1:1000
**Akt-phospho S473**		Cell Signaling Technology (#9271)	Rabbit pAb	WB: 1:1000
**Akt**		Cell Signaling Technology (#9272)	Rabbit pAb	WB: 1:1000
**Erk1/2-diphospho**		Sigma (#M8159)	Mouse mAb	WB: 1:1000
**Erk1/2**		Cell Signaling (#9102)	Rabbit pAb	WB: 1:1000
**FAK-phospho Y397**		Invitrogen (#44624G)	Rabbit pAb	WB: 1:1000
**FAK**	77	BD Bioscience #610087	Mouse mAb	WB: 1:1000
**GEF-H1-phospho S886**	E1L6D	Cell Signaling Technology (#14143)	Rabbit mAb	WB: 1:1000
**GEF-H1**		Abcam (#ab155785)	Rabbit pAb	WB: 1:1000
**MARK2**		Proteintech (#15492-1-AP)	Rabbit pAb	WB: 1:1000
**MARK2**		Cell Signaling Technology (#9118)	Rabbit pAb	WB: 1:1000
**MLC-phospho S19**		Cell Signaling Technology (#3671)	Rabbit pAb	WB: 1:500
**P115 RhoGEF**	C-19	Santa Cruz	Goat pAb	WB: 1:1000
**GAPDH**	6C5	EMD Millipore (#CB1001)	Mouse mAb	WB: 1:5000

Journal of Cell Science • Supplementary information
