# Supplementary material for: Molecular determinants of αVβ5 localization in flat clathrin lattices – role of αVβ5 in cell adhesion and proliferation
Source: J Cell Sci. 2022 Jun 6;135(11):jcs259465. doi: 10.1242/jcs.259465 (PMC9234671; doi:10.1242/jcs.259465)
Supplement: Reviewer comments [file joces-135-259465_review_history.pdf]

Journal of Cell Science

Molecular determinants of αVβ5 localization in flat clathrin lattices - role of αVβ5 in cell adhesion and proliferation

Alba Zuidema, Wei Wang, Maaike Kreft, Onno B. Bleijerveld, Liesbeth Hoekman, Jonas Aretz, Ralph Boettcher, Reinhard Faessler and Arnoud Sonnenberg
DOI: 10.1242/jcs.259465

Editor: Michael Way

Review timeline

Original submission:	13 October 2021
Editorial decision:	26 November 2021
First revision received:	20 April 2022
Accepted:	20 April 2022

Original submission

First decision letter

MS ID#: JOCES/2021/259465

MS TITLE: Integrin β5 promotes cell adhesion and proliferation via two distinct adhesion complexes

AUTHORS: Alba Zuidema, Wei Wang, Maaike Kreft, Onno Bleijerveld, Liesbeth Hoekman, Jonas Aretz, Ralph Boettcher, Reinhard Faessler, and Arnoud Sonnenberg
ARTICLE TYPE: Research Article

We have been chasing a third reviewer but given the delays we have made decision on the two reports that have come in.

To see the reviewers' reports and a copy of this decision letter, please go to: https://submit-jcs.biologists.org and click on the 'Manuscripts with Decisions' queue in the Author Area. (Corresponding author only has access to reviews.)

As you will see, the reviewers raise a number of questions that prevent me from accepting the paper at this stage. They suggest, however, that a revised version might prove acceptable, if you can address their concerns. I think you might want to consider changing the title as reviewer 2 also indicated the abstract and title does not accurately reflect the paper. If you think that you can deal satisfactorily with the criticisms on revision, I would be pleased to see a revised manuscript.

We are aware that you may be experiencing disruption to the normal running of your lab that makes experimental revisions challenging. If it would be helpful, we encourage you to contact us to discuss your revision in greater detail. Please send us a point-by-point response indicating where you are able to address concerns raised (either experimentally or by changes to the text) and where you will not be able to do so within the normal timeframe of a revision. We will then provide further guidance. Please also note that we are happy to extend revision timeframes as necessary.

Please ensure that you clearly highlight all changes made in the revised manuscript. Please avoid using 'Tracked changes' in Word files as these are lost in PDF conversion.

© 2022. Published by The Company of Biologists under the terms of the Creative Commons Attribution License (https://creativecommons.org/licenses/by/4.0/).

I should be grateful if you would also provide a point-by-point response detailing how you have dealt with the points raised by the reviewers in the 'Response to Reviewers' box. Please attend to all of the reviewers' comments. If you do not agree with any of their criticisms or suggestions please explain clearly why this is so.

Reviewer 1

Advance summary and potential significance to field

aVb5 integrin distribution differs from other integrin family members in that it is enriched in flat clathrin lattices (FCL) in comparison to other integrin subtypes that localise to focal adhesions (FA). Also, whilst aVb5 where thought to have a primary role in stabilising adhesion during mitosis, other integrins would coordinate the contractile behaviour of cells. The submitted manuscript is essentially a follow up paper of their previous publication in JSC (Zuidema et al 2018) and similar methods (imaging, mass spectrometry in combination with integrin domain swapping and mutational analysis) were used to shed light into regulatory mechanisms of aVb5 distribution. Novel findings were: (i) b5 integrins showed significantly higher affinity binding to Numb and ARH than binding to talin, thus contrasting the preference of b1/b3 integrins for talin; (ii) the critical involvement of phosphorylation of beta5 S759/762 residues for localisation to FCLs, the interaction with serine/threonine kinase MARK2 seemed to have an important role in this matter; (iii) an increase of intracellular tension led to a shift of b5 localisation away from FCLs to FAs; and (iv) that aVb5 is critical for cell proliferation on vitronectin irrespective of its localisation to FCLs or FAs. Overall, the experiments are sound and thoroughly quantified. The data provide a significant advance in the field and are interesting for the JCS readership.

Comments for the author

There are no major points to deal with in this well-written manuscript. However shortening some of the parts describing the NPXY data that were partly addressed already in the group's previous manuscript (Zuidema et al 2018) would provide a faster transition to the more novel data with the serine phosphorylation sites.
Moreover, I wonder whether a few experiments using microtubule disrupting/stabilising drugs could provide some additional mechanistic insight.
For one, the data suggest association of b5 with microtubule binding partners
(MARK1, GEF-H1) and second, microtubules regulate tension by affecting Rho GTPase activity (e.g. through GEF-H1).
A third point is that the title does not sound terribly attractive and does not reflect the to my opinion most interesting findings linked to the serine/threonine phosphorlyation sites.

Reviewer 2

Advance summary and potential significance to field

In this well written, carefully prepared and well controlled study the Sonnenberg laboratory have continued to investigate integrin recruitment to FLC.
They show that the distribution on b5-integrin between FAs and FLCs is very cell type dependent and is influenced by a number of factors. They identify the b5-
tail unique serine motif to be involved and present data indicative of Ser-
phosphorylation by MARK2 as a key control mechanism. I think this study addresses an important point about the relationship pf FAs and FLCs and significantly increases our understanding in this area.

Comments for the author

There are some points that the authors may want to consider:
integrin β5 cytoplasmic domain binds more strongly to ARH and Numb than to talin, and displays only very limited binding to kindlin-1/2. Furthermore, the phosphomimetic β5-S759/762E mutant promotes localization in FCLs.

© 2022. Published by The Company of Biologists under the terms of the Creative Commons Attribution License (https://creativecommons.org/licenses/by/4.0/).

In the BirA experiments, the authors detect no biotinylation of clathrin adaptors ARH and NUMB with the b5/b1 or b5/b1 chimeras, in contrast to the b5 wt. Is this also the case for clathrin or AP2-mu, established components of FCL?
The peptide pull-downs in figure 2 are a little problematic given the high Bg binding of NUMB and and ARH to the b1 scr peptide. Perhaps the authors could acknowledge this in the text? In addition, quantifications from several biological repeats of the pulldowns might help to establish the reproducibility and statistical significance of the differences indicated by the authors in the text.
Figure 3. These data are really nice an convincing. I wonder, however, if they could be supported by data showing increased b5-tail (serine) phosphorylation upon calicylin treatment?
Figure 4. is there a reason for using talin rather than vinculin staining as a FA marker here? The FA seem enlarged/elongated in the siMARK2 cells. Could this be analysed and mentioned in the text? Judging from the respresentative image the clathrin staining intensity is lower in the siMARK2 cells. Is this the case?
Does MARK2 depletion also influence the abundance of FLC not just b5 recruitment to them?
Would it be possible to demonstrate with an integrin tail kinase assay that MARK2 phosphorylates ß5-SERS ?

Minor.
Is this sentence in the intro perhaps incorrect? The data indicate that the SS/EE mutant is more in the FLC (Fig. 3E) and that MARK2 depletion reduced b5 localisation to FLC.
“MARK2 depletion substitution of two serines (S759/762) by phosphomimetic glutamates in the ß5 cytoplasmic domain and depletion of the serine protein kinase MARK2 that associates with ß5 diminishes the clustering of ß5 in FCLs. “

First revision

Author response to reviewers' comments

Reviewer 1 Comments for the author...

Q1: There are no major points to deal with in this well-written manuscript. However, shortening some of the parts describing the NPXY data that were partly addressed already in the group’s previous manuscript (Zuidema et al 2018) would provide a faster transition to the more novel data with the serine phosphorylation sites.

Response: We agree with the reviewer that part on the NPXY data in the current study has been addressed in our previous manuscript. We have shortened this part and merged it with that about the unique interactors and sequence of ß5 (page 6-7).

Q2: Moreover, I wonder whether a few experiments using microtubule disrupting/stabilising drugs could provide some additional mechanistic insight. For one, the data suggest association of b5 with microtubule binding partners (MARK1, GEF-H1) and second, microtubules regulate tension by affecting Rho GTPase activity (e.g. through GEF-H1).

Response: We thank the reviewer for these suggestions. We now present a series of experiments aimed at exploring the role of microtubules and the microtubule-related proteins, MARK2 and GEF-H1, in regulating the subcellular localization of integrin ß5 (Fig.4,5 and S4). We show that the disruption of microtubules, which is associated with the activation GEF-H1 and higher pMLC levels, leads to a shift in the localization of ß5 from FCLs to FAs. Furthermore, we have explored the possibility that in PA-JEB/ß4 and PA-JEB keratinocytes MARK2 promotes ß5 localization into FCLs through phosphorylation of GEF-H1 at S886. Surprisingly, we found that rather than decreasing GEF-H1 phosphorylation, the downregulation of MARK2 increased GEF-H1 phosphorylation in both PA-JEB and PA-JEB/ß4 keratinocytes. Depletion of MARK2 also increased GEF-H1 phosphorylation in calyculin A-treated PA-JEB/ß4 keratinocytes, while in the calyculin A-treated PA-JEB keratinocytes it caused a reduction in GEF-H1 phosphorylation (compared to the control calyculin A-treated PA-JEB keratinocytes). We have discussed these findings in the appropriate section (page 13-14).

© 2022. Published by The Company of Biologists under the terms of the Creative Commons Attribution License (https://creativecommons.org/licenses/by/4.0/).

Q3: A third point is that the title does not sound terribly attractive and does not reflect the to my opinion most interesting findings linked to the serine/threonine phosphorylation sites.

Response: We have now changed the title and abstract to better emphasize the interesting and novel findings (page 1-2).

Reviewer 2 Comments for the author...

There are some points that the authors may want to consider:
integrin β5 cytoplasmic domain binds more strongly to ARH and Numb than to talin, and displays only very limited binding to kindlin-1/2. Furthermore, the phosphomimetic β5-S759/762E mutant promotes localization in FCLs. In the BirA experiments, the authors detect no biotinylation of clathrin adaptors ARH and NUMB with the b5/b1 or b5/b1 chimeras, in contrast to the b5 wt. Is this also the case for clathrin or AP2-mu, established components of FCL?

Response: We thank the reviewer for his/her useful remark. In our previous work (Zuidema et al 2018) we have shown that β5, but not the β5/β1 and β5/β3 chimeras, is co-distributed with clathrin. We also reported the identification of AP2 and its binding partner, EPS15L as proximity interactors of β5 (BioID data) in that study. To confirm the association of β5 with EPS15L, we blotted the proteins that were biotinylated by β5-BirA, β5/β1-BirA and β5/β 3-BirA on a membrane and probed the membrane with antibodies against EPS15L. As shown in the formatted PDF of the response EPS15L associates with all three BirA fusion proteins, albeit more strongly with β5 that with the β5/β1 and β5/β3 chimeras.

The peptide pull-downs in figure 2 are a little problematic given the high Bg binding of NUMB and and ARH to the b1 scr peptide. Perhaps the authors could acknowledge this in the text? In addition, quantifications from several biological repeats of the pulldowns might help to establish the reproducibility and statistical significance of the differences indicated by the authors in the text.

Response: We apologize for having presented a wrongly vertically sliced blot image. The control lane with the scrambled Itg. β1 (Itg. β1 scr.) peptide actually showed the blots for Itg. β1 (+ 9 aa). We have now replaced it with the correct Itg. β1 scr. control lane (new Fig.2C), which shows hardly any background binding of the β1 scrambled peptide to ARH. Although some background binding of NUMB to the β1 scrambled peptide can still be detected, it is clear NUMB binds considerably more strongly to the β5 and β5 (Δ8 aa) peptides. For your information, we show the complete blot in the formatted PDF of the response

Figure 3. These data are really nice an convincing. I wonder, however, if they could be supported by data showing increased b5-tail (serine) phosphorylation upon calicylin treatment?

Response: We thank the reviewer for his/her remark. We have tried several times to validate phosphorylation of integrin β5 at S759/762 by mass spec, but have been unsuccessful in detecting the phosphorylation of these serine residues. Specifically, we assessed whether phosphorylation of β5 at S759 and S762 could be demonstrated in PA-JEB/β4 keratinocytes treated with 25 nM calyculin for 20 min. Cell lysates prepared from the Calyculin A-treated keratinocytes were immunoprecipitated with an anti-β5 antibody and the precipitated β5 proteins separated by gel electrophoresis followed by in-gel digestion with endolysin and liquid tandem MS/MS. Because MARK2 co-precipitated with β5, we also performed a kinase assay with the immunoprecipitated proteins and subjected the reaction mixture to MS. In both type of experiments no phosphorylation of β5 at S759 and S762 could be detected (discussed in the middle of page 13).

Figure 4. is there a reason for using talin rather than vinculin staining as a
FA marker here? The FA seem enlarged/elongated in the siMARK2 cells. Could this be analysed and mentioned in the text? Judging from the respresentative image, the clathrin staining intensity is lower in the siMARK2 cells. Is this the case? Does MARK2 depletion also influence the abundance of FLC not just b5 recruitment to them?

Response: We apologize for the inconsistent use of the FA markers. We have performed the experiment with vinculin staining and replaced the old data (Fig.4E,G). The trend of vinculin-probed data is consistent with talin-probed data. For the issue of clathrin staining intensity, we

© 2022. Published by The Company of Biologists under the terms of the Creative Commons Attribution License (https://creativecommons.org/licenses/by/4.0/).

quantified the intensity of FCLs after MARK2 depletion and found it was slightly higher than in the control group. We have now replaced the image in Fig. 2F with a more representative image of clathrin staining in the control group.

Would it be possible to demonstrate with an integrin tail kinase assay that MARK2 phosphorylates ß5-SERS ?

Response: See our response above.

Minor.
Is this sentence in the intro perhaps incorrect? The data indicate that the SS/EE mutant is more in the FLC (Fig. 3E) and that MARK2 depletion reduced b5 localisation to FLC.
"MARK2 depletion substitution of two serines (S759/762) by phosphomimetic glutamates in the ß5 cytoplasmic domain and depletion of the serine protein kinase MARK2 that associates with ß5 diminishes the clustering of ß5 in FCLs.

Response: We apologize for the mistake we made in this sentence. It has been corrected in the abstract.

Second decision letter

MS ID#: JOCES/2021/259465

MS TITLE: Molecular determinants of ß5 localization in flat clathrin lattices: Role of ß5 in cell adhesion and proliferation

AUTHORS: Alba Zuidema, Wei Wang, Maaike Kreft, Onno Bleijerveld, Liesbeth Hoekman, Jonas Aretz, Ralph Boettcher, Reinhard Faessler, and Arnoud Sonnenberg
ARTICLE TYPE: Research Article

I am happy to tell you that your manuscript has been accepted for publication in Journal of Cell Science, pending standard ethics checks.

© 2022. Published by The Company of Biologists under the terms of the Creative Commons Attribution License (https://creativecommons.org/licenses/by/4.0/).
